# Evaluation of the Good Review Practices of Countries Participating in the Southern African Development Community: Alignment and Strategies for Moving Forward

**DOI:** 10.3389/fmed.2021.742181

**Published:** 2021-08-27

**Authors:** Tariro Sithole, Gugu Mahlangu, Velma Capote, Tania Sitoie, Saren Shifotoka, Johannes Gaeseb, Silverani Padayachee, Tohlang Sehloho, Akida Khea, Adam Fimbo, Zuma Munkombwe, Bernice Mwale, Sam Salek, Stuart Walker

**Affiliations:** ^1^School of Life and Medical Sciences, University of Hertfordshire, Hatfield, United Kingdom; ^2^Medicines Control Authority of Zimbabwe, Harare, Zimbabwe; ^3^National Directorate of Pharmacy, Mozambique Ministry of Health, Maputo, Mozambique; ^4^Namibia Medicines Regulatory Council, Namibia Ministry of Health and Social Services, Windhoek, Namibia; ^5^South African Health Products Regulatory Authority, Pretoria, South Africa; ^6^Tanzania Medicines and Medical Devices Authority, Dodoma, Tanzania; ^7^Zambia Medicines Regulatory Authority, Lusaka, Zambia; ^8^Institute for Medicines Development, Cardiff, United Kingdom; ^9^Centre for Innovation in Regulatory Science, London, United Kingdom

**Keywords:** South African Development Community, ZaZiBoNa, regulatory reliance, good review practices, constrained resources

## Abstract

**Introduction:** National medicines regulatory agencies are faced with challenges including limited resources and technical capacity, resulting in countries collaborating and sharing resources to improve efficiency of the review process to facilitate access to quality-assured medicines by their populations. One such collaboration is the Southern African Development Community (SADC) medicines registration collaborative initiative, ZaZiBoNa. Countries participate in the initiative by contributing to regulatory reviews and good manufacturing practices inspections. The aim of this study was to review and compare the registration processes of regulatory authorities of Mozambique, Namibia, South Africa, Tanzania, Zambia, and Zimbabwe to identify strategies for better alignment.

**Methods:** A senior member of the division responsible for issuing marketing authorisations completed an established and validated questionnaire, which standardises the review process, allowing key milestones, activities and practices of the six regulatory authorities to be identified and compared. The completed questionnaires were validated by the heads of the respective agencies.

**Results:** The six countries vary in population and in the size of their respective regulatory agency and the resources allocated to regulatory reviews. The review processes of the six agencies were similar; however, differences were noted in the milestones recorded; for example, two of the countries did not record the start of the scientific assessment. Additionally, decisions for marketing authorisation were made by an expert committee in four of the countries and by the head of the agency and the Minister of Health in two countries. All six agencies implemented the majority of good review practices; however, the need for improvement in the areas of transparency and communication and quality decision making practices was a common finding for all six countries.

**Conclusions:** Participation in the ZaZiBoNa initiative has improved the way in which the six agencies perform regulatory reviews in their countries, highlighting the realisation of one of the key objectives of the initiative, which was building the expert capacity of member countries. Other agencies in the SADC region and beyond can use the results of this study to identify best practices, which in turn, could improve their regulatory performance.

## Introduction

The Southern African Development Community (SADC) is made up of 16 countries; Angola, Botswana, Comoros Islands, Democratic Republic of Congo, Lesotho, Madagascar, Malawi, Mauritius, Mozambique, Namibia, Seychelles, South Africa, Swaziland, United Republic of Tanzania, Zambia, and Zimbabwe (1). Although the countries have differing capacities to regulate medicines (2), they share the common challenge of inadequate capacity to review applications for medicines, resulting in backlogs and delayed access to medicines by patients. This led to the formation of a collaborative medicines registration initiative called ZaZiBoNa by four countries, Zambia, Zimbabwe, Botswana, and Namibia with technical support from the World Health Organization (WHO) Prequalification team in 2013 (3). The initiative was formally endorsed by the SADC Ministers of Health in 2015, and member states who signed the memorandum of agreement to participate in the initiative were assigned active or non-active status, depending on their capacity to conduct assessments and good manufacturing practices (GMP) inspections. The remaining countries, Mauritius and Lesotho, participate as observers (3).

### Operational Aspects of ZaZiBoNa

**Figure 1:**
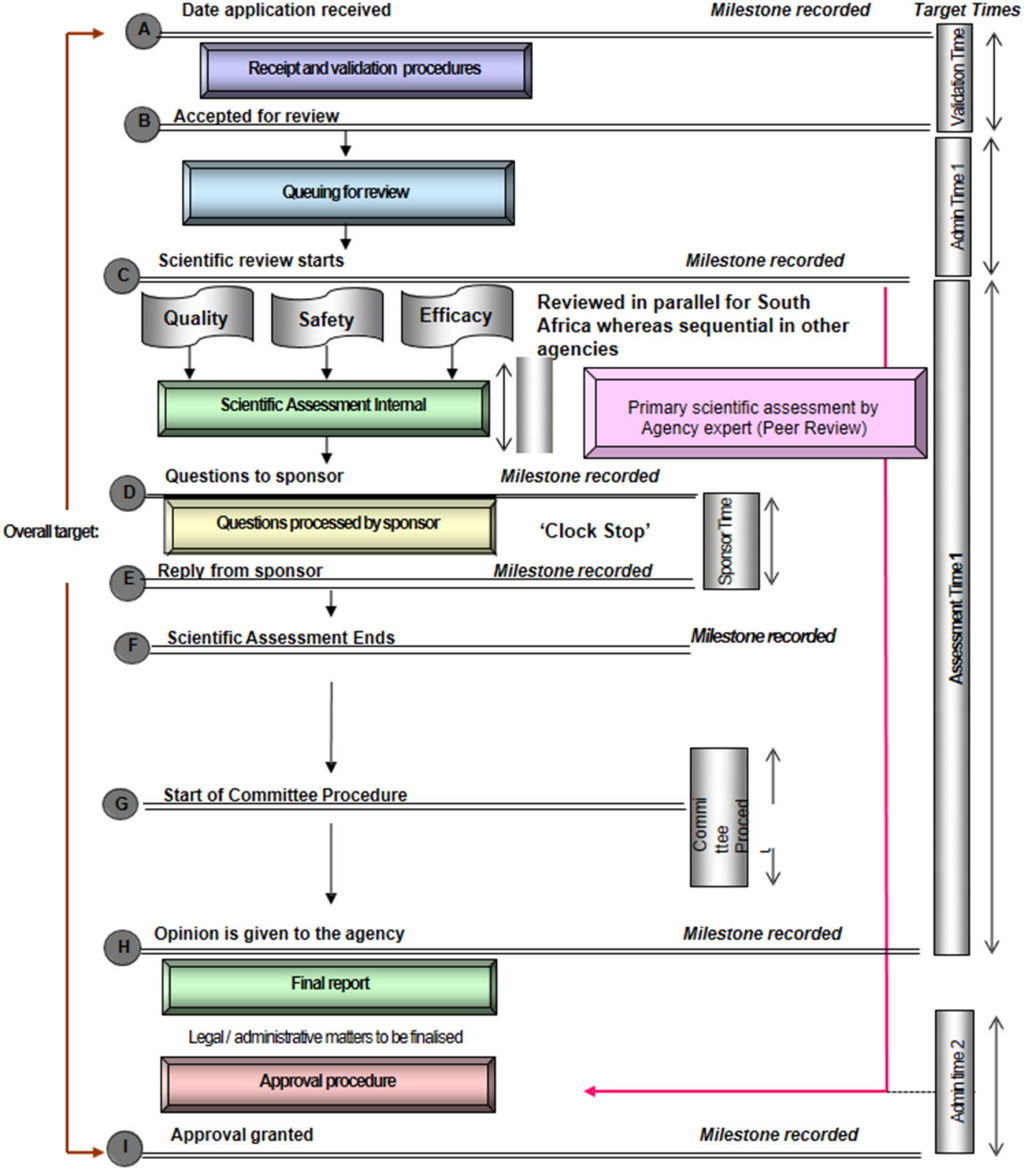
Standardised review process map for regulatory agencies. This map represents the review and authorization of a product that goes to approval after one review cycle. It should be noted that in some countries milestone G (committee procedure) may come before milestone D (questions to the applicant).

ZaZiBoNa is a SADC work-sharing initiative, in which regulatory authorities conduct joint or shared reviews of applications for registration of medicines submitted to participating countries with the applicant's consent (3). One of the successes of the ZaZiBoNa initiative is that since its inception in 2013, more than 300 products have been reviewed and the median time to a recommendation was shorter than that achieved by individual participating countries using the national procedure (4). However, because the ZaZiBoNa initiative is not a legally constituted regulatory authority, it relies significantly on the participating countries to achieve a number of key milestones in the review process, particularly those of an administrative nature (Figure 1) (3). As a result, one of the challenges that has been identified with this initiative is the fact that differences in country review processes result in questions to applicants for the same product being sent at different times by the countries, affecting registration timelines and negating the benefit of simultaneous access to various markets. Sithole et al. therefore recommended that the regulatory review processes in the individual participating countries be reviewed and the outcomes compared (3, 5). The aim of this study therefore was to review and compare the registration processes of regulatory authorities of Mozambique, Namibia, South Africa, Tanzania, Zambia, and Zimbabwe to develop recommendations for better alignment, while presenting an opportunity for the countries to learn from each other and enhance their regulatory review and patients' access to life-saving medicines. This article, the first in a two-part series, details the findings, focusing on the review processes and good review practices. The second article will address review models and metrics of process.

## Materials and Methods

### Study Participants

Nine countries with active member status in the ZaZiBoNa initiative were invited to participate in the study following a face-to-face presentation. Active member status is defined as “the capacity to conduct assessments and GMP inspections.” One of the countries (Botswana) could not complete the questionnaire because their agency had only recently been established and the lack of participation by two countries (the Democratic Republic of Congo and Malawi) was likely because of disruptions caused by the Covid-19 pandemic. Therefore, the six regulatory agencies included in this study were the National Directorate of Pharmacy in the Mozambique Ministry of Health; Namibia Medicines Regulatory Council (NMRC) in the Namibia Ministry of Health and Social Services; the South African Health Products Regulatory Authority (SAHPRA); the Tanzania Medicines and Medical Devices Authority (TMDA); the Zambian Medicines Regulatory Authority (ZAMRA); and the Medicines Control Authority of Zimbabwe (MCAZ).

### Data Collection

Each of the six agencies completed an established and validated questionnaire (6) in 2020, which described the organisational structure, the regulatory review system for market authorisation for new active substances (NASs) and generics as well as their overall review times from the date of application to the date of approval, good review practices (GrevP) and quality decision-making practices. The questionnaire allowed for the collection of data in a standardised format, enabling comparison and analyses of information collected from the six agencies.

The questionnaire consists of five parts: *Part 1*, documents the structure, organisation and resources of the agency; *Part 2*, identifies different types of review model (s) used for the scientific assessment of medicines; *Part 3*, documents information on the key milestone dates and the process using a standardised process map; *Part 4*, records how quality is built into the regulatory process (GrevP), and *Part 5*, explores the quality of the decision-making practices of the agency.

### Ethics Committee Approval

The study was approved by the Health, Science, Engineering and Technology ECDA, University of Hertfordshire, United Kingdom [Reference Protocol number: LMS/PGR/UH/04350].

## Results

For the purpose of clarity, the results of this article (first of a series of two) will be presented in four parts: Part I – organisation of the regulatory authorities; Part II – key milestones in the review process; Part III – good review practices; and Part IV – quality decision-making practices. The second article of the series will address the remaining results of the questionnaire.

### Part I - Organisation of the Regulatory Authorities

The six countries, Mozambique, Namibia, South Africa, Tanzania, Zambia, and Zimbabwe, vary in population and size of their respective regulatory agency (Table 1). South Africa (58.8 million) and Tanzania (58.6) have the largest populations, while Namibia has the smallest (2.6). Four countries, South Africa, Tanzania, Zambia, and Zimbabwe have autonomous agencies independent of the Ministry responsible for Health. All six agencies have the common mandate to regulate medicinal products, medical devices and *in vitro* diagnostics for human and veterinary use, except for Mozambique, which does not regulate products for veterinary use. In addition, the South African agency also has the mandate to control the development and use of radiation procedures (for medical use).

**Table 1:** Comparison of the country population, size of agencies and workload in 2019.

**Country**	**Mozambique**	**Namibia**	**South Africa**	**Tanzania**	**Zambia**	**Zimbabwe**
Population (millions)	29.5	2.6	58.8	58.6	17.4	16.2
Agency staff	83	26	170	103	120	143
Staff per million residents	2.8	10	2.9	1.8	6.9	8.8
Number of internal reviewers	5	4	57	45	19	18
Reviewers in agency staff	6%	15%	34%	44%	16%	13%
Total applications received	208	146	N/A^*^	873	585	203
Number of applications per reviewer	42	37	N/A^*^	19	31	11

* *SAHPRA was unable to provide data for 2019 due to mitigating circumstances related to the unfit status of the organisation's premises*.

The ratio of total staff per million residents varied across the six countries, with Namibia having the highest ratio of 10, followed by Zimbabwe at 8.8, Zambia at 6.9, South Africa at 2.9, Mozambique at 2.8, and Tanzania at 1.8. The professional background of the agency reviewers was primarily pharmacy for all six agencies and only South Arica and Tanzania had physicians as part of their review teams. Tanzania had the highest proportion of reviewers to total agency staff (44%), followed by South Africa (34%), Zambia (16%), Namibia (15%), Zimbabwe (13%), and Mozambique (6%). The agencies in South Africa, Tanzania and Zambia made use of external experts in the review of applications for registration, employing at the time of the study, 32, 36, and 8 external reviewers, respectively, while the other countries used only internal experts. Zimbabwe, however, had a provision for use of external experts even though none were employed at the time of the study.

If, hypothetically, all new applications received in a year were reviewed in that same year, then the workload; that is, the number of dossiers to be reviewed per year per internal reviewer for 2019 was the highest for Mozambique (42), followed by Namibia (37), Zambia (31), Tanzania (19), and Zimbabwe (11).The workload for South Africa could not be calculated as the agency was unable to provide data for products in 2019 due to mitigating circumstances related to the unfit status of the organisation's premises. However, all six agencies reported that they had a backlog of pending applications, therefore not all applications were reviewed in the year that they were received. The analysis also did not take into account the type of review to be conducted, the competence of reviewers or other work such as post-approval variations. It should be noted that in some of the countries due to low numbers of staff, the same reviewer was responsible for reviewing the quality, pre-clinical and clinical. The countries with greater numbers of reviewers had one reviewer focusing on quality and different reviewers for non-clinical and clinical.

#### Source of Funding

The Namibian agency was funded entirely by its government, in Mozambique the greater proportion of agency funding was from its government and a small percentage from other sources, in South Africa, 70% of agency funding was provided by its government and 30% from fees, in Tanzania, 12% of agency funding was by its government, 76% from fees and 12% from other sources, in Zambia, 95% of agency funding came from fees, and 5% from other sources and the Zimbabwe agency was funded entirely from fees. There is a significant range of fees applied for the reviews of the products, depending on their category such as new chemical entities, biologicals or generics. It is worth noting that none of the agencies charged fees for scientific advice given to applicants.

Namibia charged the lowest fees (333 USD) for new chemical entities, while South Africa charged the highest (3,558 USD) (Table 2). For biologicals, Namibia charged the lowest fees (333 USD) while Tanzania charged the highest (3,500 USD). For generics, Namibia charged the lowest fees (333 USD), while Zimbabwe charged the highest (2,500 USD). The agencies funded largely or entirely by government charged the lowest fees, whilst those relying on fees charged higher amounts with the exception of South Africa which received 70% of its budget from the Government, but charged fees comparable to Tanzania, Zambia, and Zimbabwe agencies, which are funded largely through fees.

**Table 2:** Comparison of fees charged and source of funding in 2019.

**Country**	**Mozambique**	**Namibia**	**South Africa**	**Tanzania**	**Zambia**	**Zimbabwe**
Source of funding^*^	Large % government,	100% government	70% government	12% government;	95% fees	100% fees
	small % other sources		30% Fees	76% fees; 12% other	5% other	
Fees for review of a new chemical entity (USD)	360	333	3,558	2,000	2,800	3,000
Fees for review of biologicals (USD)	360	333	2,833	3,500	2,800	3,000
Fees for review of generics (USD)	350	333	1,781	2,000	2,000	2,500

* *Actual percentages vary year to year*.

### Part II – Key Milestones in the Review Process

A standardised process map for the review and approval of medicines is shown in Figure 1. This is a simplified representation of the key milestones that are typically recorded and monitored in the review of applications in a mature regulatory system. The process map represents the review and authorisation of a product that goes to approval after one review cycle; however, in practice it usually takes more than one cycle for a medicine to be approved, some agencies limit the number of review cycles and opportunities given to applicants to respond to questions.

#### Receipt and Validation Procedures

All six agencies validated applications received for completeness in line with the applicable guidelines and statutory fees and all six agencies recorded these two milestones. At this stage, the pathway for review was determined; that is, either verification, abridged or full review. Applications that passed validation were placed in a queue awaiting scientific assessment. Incomplete applications were removed from the queue and communication was made to the applicant to provide the missing information.

#### Queue Time

The queue time is the time between the completion of validation/acceptance for review of an application and the start of the scientific assessment. This milestone was recorded by all six agencies.

#### Primary Scientific Assessment

The start of the primary scientific assessment was recorded by four of the six agencies, namely Mozambique, South Africa, Tanzania, and Zimbabwe.

#### Questions to Applicants

All six agencies collected questions into a single batch after each cycle of scientific assessment and sent these to the applicant. This time is also referred to as “clock stop” or company time, when the assessment is paused and the applicant given an opportunity to respond to queries.

#### Review by Expert Committees

Five agencies made use of a panel of external experts known as the expert committee during the review process with the agency staff serving as the secretariat, with the exception of Mozambique. The expert committee was involved after questions had been sent to applicants in some agencies and in the other agencies, questions were only sent to applicants after the committee procedure. The external committees are referred to by different names in each of the agencies; however, their function is similar. Namibia, South Africa, Zambia, and Zimbabwe were mandated to follow the Expert Committee's opinion on a product and the Committee had the responsibility for the marketing authorisation decision. For Tanzania, the Committee made a recommendation, although the final decision was made by the Director General. The decision for marketing authorisation in Mozambique was made by the Minister of Health.

#### Authorisation Procedure

Once an opinion or decision had been made on an application for marketing authorisation, there was an administrative step to finalise reports and update the labelling before the issuance of the marketing authorisation. This step was performed in all six countries.

### Part III – Good Review Practices

For the purpose of clarity, GRevPs are presented under four categories: quality measures; transparency and communications; continuous improvement initiatives; and training and education.

#### Quality Measures

The quality measures evaluated in this comparative study are listed in Table 3. Tanzania, Zambia and Zimbabwe implemented all eight quality measures while the remaining three countries (South Africa, Namibia, and Mozambique) implemented six of the eight quality measures. Apart from Mozambique, five agencies made use of expert scientific committees as well as implementing a good review practice system (formally or informally). All of the six agencies had standard operating procedures and assessment templates in place. The assessment reports were prepared in English by five agencies; whereas Mozambique prepared their reports in Portuguese, their official language. An internal quality policy was implemented by all agencies apart from Namibia. Four agencies had dedicated quality departments, apart from Namibia and South Africa, although South Africa has now appointed a quality manager with a view to establishing a dedicated quality department. All six agencies conducted peer review of assessment reports.

**Table 3:** Comparison of the quality measures implemented by the six regulatory authorities.

**Quality measure**	**Regulatory authority**					
	**Mozambique (6/8)**	**Namibia (6/8)**	**South Africa (6/8)**	**Tanzania (8/8)**	**Zambia (8/8)**	**Zimbabwe (8/8)**
Good review practice system	✘	✓^a^	✓	✓^a^	✓	✓^a^
Internal quality policy	✓	✘	✓	✓	✓	✓
Standard operating procedures for guidance of assessors	✓	✓	✓	✓	✓	✓
Assessment templates	✓	✓	✓	✓	✓	✓
Peer review (internal)	✓	✓	✘	✓	✓	✓
Dedicated quality department	✓	✘	✘^b^	✓	✓	✓
Scientific Committee	✘	✓	✓	✓	✓	✓
Shared and joint reviews	✓	✓	✓	✓	✓	✓

a *Implemented but not formally documented.*

b *A Quality Manager has now been appointed with a view to establishing a dedicated department*.

#### Transparency and Communication

Transparency in the review process improves stakeholders' (applicants as well as other stakeholders such as local agents (which may be different from applicants), wholesalers, customers who are potential applicants, ministry of health or the patients) confidence in the system. It also assists the pharmaceutical industry in preparing submissions and planning product launch dates. Transparency saves a regulatory agency time and effort as the industry would be able to access information and requirements independently.

All six agencies assigned high priority to transparency with stakeholders. Nine best practices in transparency and communication with stakeholders were evaluated and used for this comparison (Table 4). All agencies had official guidelines and lists of approved products, which were made available to the industry through their websites. Five of the agencies did not provide post-approval feedback to applicants on quality of submitted dossiers or publish advisory committee meeting dates apart from South Africa. Four of the agencies did not provide applicants with details of technical staff to contact during review of their application apart from Mozambique and South Africa. Four agencies did not provide pre-submission scientific advice to the pharmaceutical companies except for South Africa, which implemented this informally and Zimbabwe, which provided this only for the local industry.

**Table 4:** Comparison of the transparency and communication parameters in the six agencies.

**Quality measure**	**Regulatory authority**					
	**Mozambique (4/9)**	**Namibia (3/9)**	**South Africa (8/9)**	**Tanzania (4/9)**	**Zambia (4/9)**	**Zimbabwe (5/9)**
Post-approval feedback to applicant on quality of submitted dossiers	✘	✘	✓	✘	✘	✘
Details of technical staff to contact	✓	✘	✓	✘	✘	✘
Pre-submission scientific advice to industry	✘	✘	✓^a^	✘	✓	✓^c^
Official guidelines to assist industry	✓	✓	✓	✓	✓	✓
Industry can track progress of applications	✓	✓	✓^^a^, ^b^^	✓	✓	✓
Publication of summary of grounds on which approval was granted	✘	✘	✘	✘	✘	✘
Approval times	✘	✘	✓	✓	✘	✓
Advisory committee meeting dates	✘	✘	✓	✘	✘	✘
Approval of products	✓	✓	✓	✓	✓	✓

a *Implemented informally.*

b *Only for backlogged projects.*

c *Only for local industry*.

All six agencies allowed the industry to track progress of their applications (Table 4) via email and telephone contact; however, only Mozambique and Tanzania allowed applicants electronic access to the status of their applications under review. None of the agencies shared the full assessment report with applicants or published a summary basis of approval; however, Tanzania more recently has put in place a procedure for publishing public assessment reports and these should be available in 2021. All six agencies shared a list of questions after assessment and reasons for refusal with the applicant. Only Tanzania published approval times on their website, whereas South Africa and Zimbabwe published these in their annual performance plan and annual reports, respectively.

#### Continuous Improvement Initiatives

The continuous improvement initiatives included both internal and external quality audits, an internal tracking system, as well as reviews of assessors' and stakeholders' feedback. Tanzania and Zimbabwe implemented all of the five initiatives, while Zambia, Mozambique, and South Africa implemented four out of the five initiatives. Namibia implemented only two out the five initiatives (Table 5). Five agencies, apart from Namibia, conducted internal quality audits. Five agencies had internal tracking systems, except for Namibia. The assessors' feedback was reviewed by all six agencies; however, only Namibia, Tanzania, Zambia, and Zimbabwe reviewed stakeholders' feedback.

**Table 5:** Comparison of continuous improvement initiatives in the six regulatory authorities.

**Quality measure**	**Regulatory authority**					
	**Mozambique (4/5)**	**Namibia (2/5)**	**South Africa (5/5)**	**Tanzania (5/5)**	**Zambia (4/5)**	**Zimbabwe (5/5)**
External quality Audits	✓	✘	✓	✓	✘	✓
Internal quality Audits	✓	✘	✓	✓	✓	✓
Internal tracking Systems	✓	✘	✓^a^	✓	✓	✓
Reviews of assessors' feedback	✓	✓	✓^a^	✓	✓	✓
Reviews of stakeholders' feedback	✘	✓	✓	✓	✓	✓

a *Implemented informally with no documented system*.

#### Training and Education

The measures evaluated under training and education contribute to the development of personnel and the efficiency of the regulatory review process. These measures are induction training, on-the-job training, in-house and external courses, international workshops, placements and secondments in other regulatory authorities, post-graduate degrees, and collaboration with other agencies. All six of the regulatory authorities in this comparative study implemented all of the measures for training and education. However, four agencies had formal training programmes for assessors except for Mozambique and Namibia.

### Part IV - Quality Decision-Making Practices

The decision-making process should be routinely measured to ensure consistency and quality of decisions made in the review and approval of medicines. Three of the agencies had a framework in place that forms the basis of the decision to approve or reject applications for new medicines, namely South Africa, Tanzania, and Zambia. South Africa and Tanzania fully incorporated all of the 10 quality decision-making practices (QDMPs) developed by Donelan et al. as an aid to decision making (7) into their frameworks and these were fully adhered to in practice. Zambia incorporated six of the 10 practices into their framework and fully adhered to four. Zimbabwe did not have a documented decision-making framework, but used a decision tree approach, fully adhering to seven out of the 10 decision-making practices and partially adhering to three. Mozambique and Namibia did not have a documented quality decision-making framework. Interestingly, all six agencies stated that the decision-making process could be improved, while the two agencies without frameworks indicated their intention is to develop them by 2022.

## Discussion

The aim of this study was to evaluate the regulatory review processes of six countries in the SADC region that are active members of the ZaZiBoNa collaborative medicines registration initiative and compare the outcomes in order to identify best practices. A common finding among the six regulatory authorities was that participation in the ZaZiBoNa initiative has improved the way in which they perform regulatory reviews in their countries, and this highlights how one of the key objectives of the initiative, which is to build expert capacity of member countries is being realised. In addition to identifying the differences and similarities in the processes in countries currently participating in the ZaZiBoNa initiative as active members, the results of this study will enable the regulatory authorities, the majority of which are in low-to-middle-income countries (LMICs), to benchmark processes, resources, and capacity, something which in the past was difficult due to lack of information in the public domain (8).

For industry, the results of this study provide an opportunity to better understand the regulatory review processes in the six agencies as well as the relevant challenges when planning future submissions. The commitment to continuous improvement, transparency and the desire to engage with industry shown by all the agencies, reflects a new way of doing business that should encourage further investment in terms of medicines development and regulatory submissions made to these countries and the SADC region as a whole.

Mature agencies such as Australia's Therapeutic Goods Administration and Health Canada's Health Products and Food Branch have a staff per million residents' ratio in 2021 of 31 and 60, respectively (9). In contrast, all six agencies in this study had a staff per million residents' ratio <10, confirming resource limitations faced by agencies in LMICs. In addition, a finding of this study was that there is a difference in human resources available to conduct reviews in the six agencies within the SADC region. Of note, countries with higher workloads had no targets for the scientific assessment or overall approval process, which points to overwhelmed resources. A workforce should be adequate in skill and numbers for greater operational efficiency. In addition, retention of skills after investing in staff training is of paramount importance for agencies to deliver their mandate in a timely manner.

The results of this study can be used as a baseline going forward and presents an opportunity for agencies to re-examine their processes to determine areas of improvement, particularly where another agency with a comparable workload is able to achieve shorter registration times. Routine recording of the milestones studied here will enable the monitoring and measurement of key performance indicators such as timelines for validation, queue time, scientific assessment and the overall approval, will enable the rapid identification of areas requiring improvement and a proposal of gap-closing measures such as re-engineering of processes or the injection of additional resources by the agencies.

While most of the agencies in the study indicated that resources could be optimised by placing reliance on mature agencies, there is opportunity to further reduce timelines through reliance on other agencies in the SADC region, as is already being done by one of the agencies.

Although the ZaZiBoNa collaborative medicines registration process was not directly evaluated in this study, it was possible to see the reason for the difference in time to registration among the participating countries after a recommendation for approval by ZaZiBoNa. The initiative relies on countries with differing capacities, resources and administrative processes to carry out a significant part of the review process. There is a need for a review of the current model used for the ZaZiBoNa initiative in the next strategic period to minimise the dependence on the country process and increase operational efficiency.

### Recommendations

As a result of this study several recommendations could be considered by these agencies.

1. **Performance measurement**: In order to benchmark the regulatory review process and monitor performance, agencies should consider measuring and documenting the key milestones and publishing the relevant timelines.
2. **Improvement initiatives:** Agencies could consider re-examining their processes to evaluate where they can be improved, and to learn from agencies with comparable workloads who are achieving shorter timelines.
3. **Sharing assessment reports:** Agencies participating in the ZaZiBoNa initiative should consider entering into a memorandum of understanding to share unredacted assessment reports for products that are not submitted to the initiative, which constitute the majority of the agencies' workload.
4. **Increased transparency and communication**: Agencies would benefit from implementing additional measures of transparency and communication in line with international best practices such as sharing of assessment reports with applicants and publishing approval times, advisory committee dates and a summary basis of approval.
5. **Improved performance**: Agencies should consider using the results of this study to propose the provision of adequate resources to improve timelines and patients' access to medicines.
6. **Quality decisions**: There is a need in some agencies for training and capacity building in quality decision making.
7. **ZaZiBoNa operating model**: The participating countries could consider reviewing the existing operational model for improved efficiency.

## Conclusions

This study incorporated fully autonomous and non-autonomous agencies with both large and small populations, providing a representative sample of SADC countries. Those countries that were not able to participate in the study and other countries in LMICs could use the results to benchmark and improve their own processes. If other agencies in the region were to evaluate their review process using the approach described in this study, they would also be able to identify best practices, which in turn could improve their regulatory performance.

## Data Availability Statement

The original contributions presented in the study are included in the article/Supplementary Material, further inquiries can be directed to the corresponding author.

## Ethics Statement

The study was approved by the Health, Science, Engineering and Technology ECDA, University of Hertfordshire [Reference Protocol number: LMS/PGR/UH/04350].

## Author Contributions

TSith, SSa, and SW contributed to the design of the study, implementation of the research, analysis of the results, and drafting of the manuscript. GM, VC, TSito, SSh, JG, SP, TSe AK, AF, ZM, and BM contributed to the implementation of the research and critical review of the manuscript. All authors contributed to the article and approved the submitted version.

## Conflict of Interest

The authors declare that the research was conducted in the absence of any commercial or financial relationships that could be construed as a potential conflict of interest.

## Publisher's Note

All claims expressed in this article are solely those of the authors and do not necessarily represent those of their affiliated organizations, or those of the publisher, the editors and the reviewers. Any product that may be evaluated in this article, or claim that may be made by its manufacturer, is not guaranteed or endorsed by the publisher.
