# Supplementary material for: Evaluation of the Good Review Practices of Countries Participating in the Southern African Development Community: Alignment and Strategies for Moving Forward
Source: Front Med (Lausanne). 2021 Aug 27;8:742181. doi: 10.3389/fmed.2021.742181 (PMC8429788; doi:10.3389/fmed.2021.742181)
Supplement: Supplementary file 1 [file Data_Sheet_1.pdf]

OpERA

Optimising Efficiencies in Regulatory Agencies

QUESTIONNAIRE

CIRS Centre for Innovation in Regulatory Science

OpERA: Optimising Efficiencies in Regulatory Agencies

ASSESSING THE REGULATORY REVIEW PROCESS IN EMERGING MARKETS

Key milestones, target times, and quality of decision making in the assessment and registration process

Please return this questionnaire to:

Neil McAuslane
nmcauslane@cirsci.org

Stuart Walker
Drstuartwalker@me.com

CONTENTS

CONTENTS 3
BACKGROUND 4
OBJECTIVES 4
OUTPUT 5
ABOUT THE QUESTIONNAIRE 5
FOCUS OF THE QUESTIONNAIRE 6
PART 1. ORGANISATION OF THE AGENCY 7
PART 2. TYPES OF REVIEW MODELS 12
PART 3. KEY MILESTONES IN THE REVIEW PROCESS 18
PART 4. GOOD REVIEW PRACTICES (GRevP): BUILDING QUALITY INTO THE REVIEW PROCESS 28
PART 5. QUALITY DECISION-MAKING PROCESSES 39
PART 6. CONCLUDING OBSERVATIONS 41
ACKNOWLEDGEMENT 41
GLOSSARY AND ABBREVIATIONS 42
APPENDIX I – QUALITY DECISION-MAKING PRACTICES 45
APPENDIX II – IN CASE OF REGIONAL ASSESSMENT .... **Error! Bookmark not defined.**

The Centre for Innovation in Regulatory Science (CIRS)

CIRS - The Centre for Innovation in Regulatory Science Limited - is a neutral, independently managed UK-based subsidiary company, forming part of Clarivate Analytics (UK) Limited. CIRS' mission is to maintain a leadership role in identifying and applying scientific principles for the purpose of advancing regulatory and HTA policies and processes. CIRS provides an international forum for industry, regulators, HTA and other healthcare stakeholders to meet, debate and develop regulatory and reimbursement policy through the innovative application of regulatory science and to facilitate access to medical products through these activities. This is CIRS' purpose. CIRS is operated solely for the promotion of its purpose. The organisation has its own dedicated management and advisory boards, and its funding is derived from membership dues, related activities, special projects and grants.

Centre for Innovation in Regulatory Science (CIRS)
Friars House, 160 Blackfriars Road, London SE1 8EZ, United Kingdom
Email: cirs@cirsci.org
Website: www.cirsci.org

Confidentiality
CIRS recognises that much of these data may be highly sensitive. CIRS has more than 20 years of experience in handling similar data provided by agencies regarding individual products in regulatory review. **All information collected from individual agencies will be kept strictly confidential. No data that will identify an individual agency will be reported or made available to any third party.** External reports or presentations of the data will include only blinded results and any appropriate analytical interpretations.

ASSESSING THE REGULATORY REVIEW PROCESS IN EMERGING MARKETS

Review of key milestones, target times and quality of decision-making in the assessment and registration process

BACKGROUND

This questionnaire supports an on-going programme by CIRS, focusing on the regulation of new medicines in emerging markets, and looking at how regulatory agencies build quality into their review process.

The first phase was initiated in January 2004 to assess the regulatory environment in some 30 countries, using comparative data, at the country and regional level, to identify the key issues for improving review practices and making new medicines available in an efficient and timely manner. Some of these, for example, the timing and use of the Certificate of a Pharmaceutical Product (CPP) and the length of the review process, were analysed in detail. This project highlighted the need to understand more about the different steps in the review process and the way in which these affect the overall timeline. Regulatory authorities also showed an interest in having a greater understanding of how agencies are building quality into the review process.

Through this on-going programme, CIRS maps the key milestones and associated activities, for each participating agency, for new marketing applications, and to identify the processes and procedures associated with the implementation of Good Review Practices (GRevP) that help build quality into the review process. This provides a platform to enable information sharing across agencies.

This questionnaire has been designed to collate information in a single place; agencies may have collected some of these data for other assessment (benchmarking) projects. However, **this project has several unique aspects**:

- It collects all the key information in a **single document** from which a consolidated Country Report will be created;
- It allows the metrics that are collected here and, in the future, to be related to the **PROCESS** that the agency uses thereby allowing for a more qualified assessment;
- It is part of a global programme called Optimising Efficiencies in Regulatory Agencies (OpERA), coordinated by CIRS on behalf of regulatory agencies around the world. The milestones and questions have been carefully crafted to be **relevant to any agency - large or small, mature or maturing - to provide relevant data that can be used for internal purposes or as applicable, for agency-to-agency comparisons**. For example, see Emel Mashaki Ceyhan et al: The Turkish Medicines and Medical Devices Agency: Comparison of Its Registration Process with Australia, Canada, Saudi Arabia, and Singapore. *Frontier's in Pharmacology* January 2018, Volume 9, Article 9.

OBJECTIVES

The objectives of this on-going programme are to:

- Identify the key milestones and target times for each agency and the main activities between milestones;
- Identify the model(s) of the review which is being undertaken by each agency;
- Identify opportunities for the exchange of better practices amongst regulatory authorities;
- Assess how agencies are building quality into the assessment and registration processes.

OUTPUT

Participating agencies will receive a Country Report derived from the data provided in this Questionnaire, with which they can compare their regulatory procedures with those of peer agencies across regions. This includes an analysis of where time is spent in the review process.

The outcome allows an analysis of the quality measures that are in place for a certain type of review, and provides a baseline for subsequent comparative studies across agencies to establish best practices.

ABOUT THE QUESTIONNAIRE

This questionnaire is divided into five sections:

Part 1: Organisation of the agency**:** The **Introduction** to the questionnaire asks the agency to provide current information on its structure, organisation and resources.

Part 2: Types of review models: Explores **review model(s)** for the **scientific assessment of medicines** in terms of the extent to which data is assessed in detail by the agency, and how the agency might rely on the results of assessments and reviews carried out elsewhere.

Part 3: Key milestones in the review process**:** This part of the questionnaire is based on the General Model, giving a process map and milestones, that has been developed from studying procedures followed in 'established' and 'emerging' regulatory agencies. It captures the main steps in the review and approval process and identifies key 'milestone' dates in the process. This allows for the analysis of timelines.

Part 4: Good Review Practices (GRevP): Building quality into the regulatory process looks at the activities that contribute to those measures that have been adopted to improve consistency, transparency, timeliness, and competency in the review processes.

Part 5: Quality Decision-Making Processes**:** This part of the questionnaire explores to the quality of the decision-making process and whether the agency has measures in place to ensure that good decisions are made around the data during the registration process.

Where appropriate, additional information may be obtained during face-to-face agency-CIRS interactions.

FOCUS OF THE QUESTIONNAIRE

This questionnaire is intended, primarily, to document procedures and practices that relate to medicines that are the subject of **major** applications; i.e., new active substances and major line extensions (see Glossary).

New Active Substance (NAS)

A new chemical, biological, or pharmaceutical active substance including:

- a chemical, biological, or radiopharmaceutical substance not previously authorised as a medicinal product;
- an isomer, mixture of isomers, a complex or derivative or salt of a chemical substance not previously authorised as a medicinal product, but differing in properties regarding safety and efficacy from that chemical substance previously authorised;
- a biological substance previously authorised as a medicinal product, but differing in molecular structure, nature of the source material or manufacturing process;
- a radiopharmaceutical substance which is radio nucleotide, or a ligand not previously authorised as a medicinal product, or the coupling mechanism to link the molecule and the radio nucleotide has not been previously authorised.

Major Line Extension (MLE)

A major line extension is a change to an authorised Medicinal Product that is sufficiently great that it cannot be considered as a simple variation to the original product, but requires a new product authorisation. Such changes include major new therapeutic indications or new disease states, extension to new patient populations (e.g., paediatrics), a new route of administration or a novel drug delivery system.

PART 1. ORGANISATION OF THE AGENCY

As background to the discussions about your agency, its practices and procedures it would be helpful to have the following basic information on its structure and the way it is organized:

Title of the Agency/Division responsible for the regulation of medicinal products for human use: Click or tap here to enter text.

If this is part of a parent agency with a wider remit (e.g., food and drugs) please give the title: Click or tap here to enter text.

About the agency

1.1 Indicate which of the following best describes this agency:

☐ Autonomous agency, independent from the Health Ministry administration
☐ Operates within the administrative structure of the Health Ministry

Date of establishment of the current agency: Click or tap here to enter text.

Scope of Activities

1.2 Please indicate the scope of responsibility of the agency:

☐ Medicinal products for human use
☐ Medicinal products for veterinary use
☐ Medical devices and in vitro diagnostics

1.3 Indicate the main activities that are covered by the agency:

☐ Marketing authorisations/product licences
☐ Clinical trial authorisations
☐ Post-marketing surveillance
☐ Regulation of advertising
☐ Laboratory analysis of samples
☐ Price regulation
☐ Other: Site inspections (site visits), GMP inspections

Budget / Funding

Please indicate whether the following data:

☐ are in the public domain
☐ should be treated as confidential

1.4 ***Please provide the following information on the agency budget for the regulation of medicinal products for human use:***

	**Local currency** (please specify: Click or tap here to enter text.)	**US$**
Total annual budget	Click or tap here to enter text.	Click or tap here to enter text.
Year for which data are given	Click or tap here to enter text.	
If the budget is sub-divided according to different activities, please specify % of total budget:		
Clinical trial authorisations	Click or tap here to enter text.	Click or tap here to enter text.
Marketing authorisations	Click or tap here to enter text.	Click or tap here to enter text.
Pharmacovigilance	Click or tap here to enter text.	Click or tap here to enter text.
Other post-marketing controls	Click or tap here to enter text.	Click or tap here to enter text.
Other activities, please specify: *Click or tap here to enter text.*	Click or tap here to enter text.	Click or tap here to enter text.

Sources of funding

1.5 *Please provide the following information in relation to the way the agency is funded:*

☐ Funded entirely by the government
☐ Self-funded entirely from fees
☐ Partially funded from different sources (please give proportions of total budget):
% Government: Click or tap here to enter text.
% Fees: Click or tap here to enter text.
% Other (please specify): Click or tap here to enter text.

Review team

Please note that the following questions refer to the regulation of **medicinal products for human use.**

1.6 *Please provide information on staff numbers:*

- Total staff in the agency: Click or tap here to enter text.
- Total number of reviewers for applications for marketing authorisations/ product licences: Click or tap here to enter text.
- Number of reviewers for applications for marketing authorisations/ product licences or synthetic and biological products: Click or tap here to enter text.

1.7 ***Please indicate the professional background and numbers of the technical agency staff assigned to the review and assessment of medicinal products:***

	**Number employed as assessors (degree/expertise)**			
	**Total**	with PhD or PharmD	with Master Degree	Other
Physicians	Click or tap here to enter text.	Click or tap here to enter text.	Click or tap here to enter text.	Click or tap here to enter text.
Statisticians	Click or tap here to enter text.	Click or tap here to enter text.	Click or tap here to enter text.	Click or tap here to enter text.
Pharmacists	Click or tap here to enter text.	Click or tap here to enter text.	Click or tap here to enter text.	Click or tap here to enter text.
Other Scientists	Click or tap here to enter text.	Click or tap here to enter text.	Click or tap here to enter text.	Click or tap here to enter text.
Project Managers	Click or tap here to enter text.	Click or tap here to enter text.	Click or tap here to enter text.	Click or tap here to enter text.

Fees charged for review applications

1.8 Are fees charged to sponsors for the review and assessment of applications for medicinal products for human use?

☐ YES
☐ NO

*1.9 If **YES**, please provide the following information:*

Marketing Authorisation Application fee for:	**Local currency (please specify:)**	**US$ (rounded)**
New Active Substance synthesis		Click or tap here to enter text.
New Active Substance biological		Click or tap here to enter text.
Established ingredient - proprietary product synthesis	Click or tap here to enter text.	Click or tap here to enter text.
Established ingredient - proprietary product biological	Click or tap here to enter text.	Click or tap here to enter text.
Generic product	Click or tap here to enter text.	Click or tap here to enter text.
Biological competitor product		Click or tap here to enter text.
Variations		Click or tap here to enter text.
Major line extension	Click or tap here to enter text.	Click or tap here to enter text.
Other (Please specify)<br>Locally manufactured generics?<br>Retention fee?<br>Fast track / Priority?<br><br>Vaccine	=	Click or tap here to enter text.
Does the agency charge a fee for scientific advice?<br>☐ YES<br>☐ NO<br>If **YES,** please provide fee →	Click or tap here to enter text.	Click or tap here to enter text.

Applications

1.10 ***Applications received***

Type	Number of applications received in each year			Current backlog
	2018	2019	2020	
New Active Substance	Click or tap here to enter text.	Click or tap here to enter text.	Click or tap here to enter text.	Click or tap here to enter text.
Major line extension	Click or tap here to enter text.	Click or tap here to enter text.	Click or tap here to enter text.	Click or tap here to enter text.
Generics (all)	Click or tap here to enter text.	Click or tap here to enter text.	Click or tap here to enter text.	Click or tap here to enter text.
WHO Pre-qualified generics (if applicable)	Click or tap here to enter text.	Click or tap here to enter text.	Click or tap here to enter text.	Click or tap here to enter text.

1.11 ***Applications determined***

Type	Number of applications determined in each year		
	2018	2019	2020
New Active Substances approved	Click or tap here to enter text.	Click or tap here to enter text.	Click or tap here to enter text.
New Active Substances refused	Click or tap here to enter text.	Click or tap here to enter text.	Click or tap here to enter text.
Major line extensions approved	Click or tap here to enter text.	Click or tap here to enter text.	Click or tap here to enter text.
Major line extension refused	Click or tap here to enter text.	Click or tap here to enter text.	Click or tap here to enter text.
Generics approved	Click or tap here to enter text.	Click or tap here to enter text.	Click or tap here to enter text.
Generics refused	Click or tap here to enter text.	Click or tap here to enter text.	Click or tap here to enter text.
WHO Pre-qualified generics approved	Click or tap here to enter text.	Click or tap here to enter text.	Click or tap here to enter text.
WHO Pre-qualified generics refused	Click or tap here to enter text.	Click or tap here to enter text.	Click or tap here to enter text.

Additional documentation

To assist CIRS to better understand your organisation, please provide copies of any *organisation charts* ***that show the structure of the agency and its relationship to other regulatory bodies; e.g., medical device agency. It would also be very useful to have copies of any background papers that describe the*** *functions*, *remit,* ***and*** *mission* ***of the agency.***

PART 2. TYPES OF REVIEW MODELS

Three basic types of scientific review have been identified. Many agencies apply a different level of data assessment to different applications, according to the type of product and/or its regulatory status with other agencies. The data assessment models for scientific review are described below and further questions are set out to analyse the types of scientific review in more detail.

Please indicate by checking the boxes below, which descriptions fit the model(s) used by your agency in the assessment of major applications i.e., new active substances (NASs) and major line extensions (MLE) as described earlier.

Data Assessment Type 1 (Verification)

This model is used to reduce duplication of effort by agreeing that the importing country will allow certain products to be marketed locally once they have been authorised by one or more recognised reference agencies, elsewhere. The main responsibility of the agency in the importing country is to 'verify' that the product intended for local sale has been duly registered as declared in the application and that the product characteristics (formulation, composition) and the prescribing information (use, dosage, precautions) for local marketing conforms to that agreed in the reference authorisation(s).

2.1 Type 1 is:

☐ Not used
☐ Used for all major applications
☐ Used for selected applications (please specify): Click or tap here to enter text.

Comment: Click or tap here to enter text.

2.2 Data requirements for Type 1 Assessments (verification) - What do you review/assess?

**CPP/Public assessment reports/un-redacted assessment reports/Free sales certificate/etc**	Click or tap here to enter text.
**Similarity to registered product**	Click or tap here to enter text.
**Quality data**	Click or tap here to enter text.
**Non-clinical data**	Click or tap here to enter text.
**Clinical data**	Click or tap here to enter text.
**Local benefit-risk assessment**	Click or tap here to enter text.

Data Assessment Type 2 (Abridged)

This model also conserves resources by not re-assessing scientific supporting data that has been reviewed and accepted elsewhere but includes an 'abridged' independent review of the product in terms of its use under local conditions. This might include a review of the pharmaceutical (CMC) data in relation to climatic conditions and distribution infrastructure and a benefit-risk assessment in relation to use in the local ethnic population, medical practice/culture and patterns of disease and nutrition.

Approval by a recognised agency elsewhere is a pre-requisite before the local authorisation can be granted but the initial application need not necessarily be delayed until formal documentation such as a Certificate of a Pharmaceutical Product (CPP) is available.

2.3 Type 2 is:

☐ Not used
☐ Used for all major applications
☐ Used for selected applications (please specify): Click or tap here to enter text.

Comment: Click or tap here to enter text.

2.4 Data requirements for Type 2 Assessments (abridged)- What do you review/assess?

**CPP/Public assessment reports/un-redacted assessment reports/Free sales certificate/etc**	Click or tap here to enter text.
**Similarity to registered product**	Click or tap here to enter text.
**Quality data**	Click or tap here to enter text.
**Non-clinical data**	Click or tap here to enter text.
**Clinical data**	Click or tap here to enter text.
**Local benefit-risk assessment**	Click or tap here to enter text.

Data Assessment Type 3 (Full)

In this model the agency has suitable resources, including access to appropriate internal and external experts, to carry out a 'full' review and evaluation of the supporting scientific data (quality, pre-clinical, clinical) for a major application. A Type 3 assessment could be carried out on a new application that has not been approved elsewhere but, in practice, legal requirements may dictate that the product must be authorised by a reference agency before the local authorisation can be finalised.

2.5 Type 3 is:

☐ Not used
☐ Used for all major applications
☐ Used for selected applications (please specify): Click or tap here to enter text.
☐ Full review conducted but product must still be authorised by a reference agency prior to final authorisation

Comment: Click or tap here to enter text.

2.6 Data requirements for Type 3 Assessments (full)- What do you review/assess?

**CPP/Public assessment reports/un-redacted assessment reports/Free sales certificate/etc**	Click or tap here to enter text.
**Similarity to registered product**	Click or tap here to enter text.
**Quality data**	Click or tap here to enter text.
**Non-clinical data**	Click or tap here to enter text.
**Clinical data**	Click or tap here to enter text.
**Local benefit-risk assessment**	Click or tap here to enter text.

Recognized reference agencies

*2.7 If your agency has **recognised 'reference agencies'** (as may be used for reliance or recognition in Types 1 and 2 reviews) please list the countries/agencies/authorities:*

Click or tap here to enter text.

Priority / fast-track products

2.8 Does your company have available:

☐ A priority review track
☐ A fast track (if different from priority)Data requirements and assessment

2.9 Please tick relevant boxes in the following table

<table>
<tr><th></th><th></th><th colspan="2">Type 1: VERIFICATION</th><th colspan="2">Type 2: ABRIDGED</th><th>Type 3: FULL REVIEW</th><th>Priority/fast track products</th></tr>
<tr><td rowspan="3">Evidence of authorisation by other authorities</td><td>Requirements for a CPP as part of the review</td><td colspan="2">☐ with application
☐ before authorisation
☐ not essential</td><td colspan="2">☐ with application
☐ before authorisation
☐ not essential</td><td>☐ with application and before local authorisation
☐ not essential
☐ if available at the time of submission</td><td>☐ with application
☐ before authorisation
☐ not essential</td></tr>
<tr><td>Other documentation from the authorising agencies accepted as evidence of registration</td><td colspan="2">☐ letter of authorisation
☐ copy of full authorisation
☐ Internet evidence</td><td colspan="2">☐ letter of authorisation
☐ copy of full authorisation
☐ Internet evidence</td><td>☐ letter of authorisation
☐ copy of full authorisation
☐ Internet evidence
☐ None</td><td>☐ letter of authorisation
☐ copy of full authorisation
☐ Internet evidence
☐ None</td></tr>
<tr><td>Other evidence accepted</td><td colspan="2">Click or tap here to enter text.</td><td colspan="2">Click or tap here to enter text.</td><td>Click or tap here to enter text.</td><td>Click or tap here to enter text.</td></tr>
<tr><td rowspan="10">Verification of identity between the authorised product and the local application</td><td></td><td colspan="2">Type 1 VERIFICATION</td><td colspan="2">Type 2 ABRIDGED</td><td>Type 3 FULL</td><td></td></tr>
<tr><td>Information must be:</td><td>Identical</td><td>Closely similar</td><td>Identical</td><td>Closely similar</td><td>Not applicable</td><td></td></tr>
<tr><td>Dosage form</td><td>☐</td><td>☐</td><td>☐</td><td>☐</td><td></td><td></td></tr>
<tr><td>Strength</td><td>☐</td><td>☐</td><td>☐</td><td>☐</td><td></td><td></td></tr>
<tr><td>Ingredients</td><td>☐</td><td>☐</td><td>☐</td><td>☐</td><td></td><td></td></tr>
<tr><td>Indications and dosage</td><td>☐</td><td>☐</td><td>☐</td><td>☐</td><td></td><td></td></tr>
<tr><td>Warnings and precaution</td><td>☐</td><td>☐</td><td>☐</td><td>☐</td><td></td><td></td></tr>
<tr><td>Product label</td><td>☐</td><td>☐</td><td>☐</td><td>☐</td><td></td><td></td></tr>
<tr><td>Product name</td><td>☐</td><td>☐</td><td>☐</td><td>☐</td><td></td><td></td></tr>
<tr><td>Other (specify)</td><td>☐</td><td>☐</td><td>☐</td><td>☐</td><td></td><td></td></tr>
</table>

**Scientific data required to support the application (Reference is made below to sections of the ICH Common Technical Document (CTD) as an example of the level of detail but does not imply that the CTD in necessarily accepted**		**Type 1**<br>VERIFICATION	**Type 2**<br>ABRIDGED	**Type 3**<br>FULL	**Priority/fast track products**
	Pharmaceutical quality/CMC	☐ Summary data (Mod 2.3)<br>☐ Summary + full stability<br>☐ Full data (Mod 3)	☐ Summary data (Mod 2.3)<br>☐ Summary + full stability<br>☐ Full data (Mod 3)	☐ Summary data (Mod 2.3)<br>☐ Summary + full stability<br>☐ Full data (Mod 3)	☐ Summary data (Mod 2.3)<br>☐ Summary + full stability<br>☐ Full data (Mod 3)
	Non-clinical data	☐ Written summary (Mod 2.4)<br>☐ Tabulated data (Mod 2.5)<br>☐ Full data (Mod 4)	☐ Written summary (Mod 2.4)<br>☐ Tabulated data (Mod 2.5)<br>☐ Full data (Mod 4)	☐ Written summary (Mod 2.4)<br>☐ Tabulated data (Mod 2.5)<br>☐ Full data (Mod 4)	☐ Written summary (Mod 2.4)<br>☐ Tabulated data (Mod 2.5)<br>☐ Full data (Mod 4)
	Clinical data	☐ Written summary (Mod 2.5)<br>☐ Tabulated data (Mod 2.6)<br>☐ Full data (Mod 5)	☐ Written summary (Mod 2.5)<br>☐ Tabulated data (Mod 2.6)<br>☐ Full data (Mod 5)	☐ Written summary (Mod 2.5)<br>☐ Tabulated data (Mod 2.6)<br>☐ Full data (Mod 5)	☐ Written summary (Mod 2.5)<br>☐ Tabulated data (Mod 2.6)<br>☐ Full data (Mod 5)
**Extent of Scientific Review**		**Type 1**	**Type 2**	**Type 3**	**Priority/fast track products**
	Quality/CMC data	☐ Only examined if there is a query<br>☐ 'Check list' review for completeness of data<br>☐ Selective review in detail (e.g. stability, specification)<br>☐ Detailed assessment and evaluation report	☐ Only examined if there is a query<br>☐ 'Check list' review for completeness of data<br>☐ Selective review in detail (e.g. stability, specification)<br>☐ Detailed assessment and evaluation report	☐ Only examined if there is a query<br>☐ 'Check list' review for completeness of data<br>☐ Selective review in detail (e.g. stability, specification)<br>☐ Detailed assessment and evaluation report	☐ Only examined if there is a query<br>☐ 'Check list' review for completeness of data<br>☐ Selective review in detail (e.g. stability, specification)<br>☐ Detailed assessment and evaluation report
	Comments:	Click or tap here to enter text.	Click or tap here to enter text.	Click or tap here to enter text.	Click or tap here to enter text.

	Non-clinical data	☐ Only examined if there is a query<br>☐ 'Check list' review for completeness of data<br>☐ Detailed assessment and evaluation report	☐ Only examined if there is a query<br>☐ 'Check list' review for completeness of data<br>☐ Detailed assessment and evaluation report	☐ Only examined if there is a query<br>☐ 'Check list' review for completeness of data<br>☐ Detailed assessment and evaluation report<br>☐ Not at all	☐ Only examined if there is a query<br>☐ 'Check list' review for completeness of data<br>☐ Detailed assessment and evaluation report
	Comments:	Click or tap here to enter text.	Click or tap here to enter text.	Click or tap here to enter text.	Click or tap here to enter text.
	Clinical data	☐ Only examined if there is a query<br>☐ 'Check list' review for completeness of data<br>☐ Selective review in detail (e.g. stability, specification)<br>☐ Detailed assessment and evaluation report	☐ Only examined if there is a query<br>☐ 'Check list' review for completeness of data<br>☐ Selective review in detail (e.g. stability, specification)<br>☐ Detailed assessment and evaluation report	☐ Only examined if there is a query<br>☐ 'Check list' review for completeness of data<br>☐ Selective review in detail (e.g. stability, specification)<br>☐ Detailed assessment and evaluation report	☐ Only examined if there is a query<br>☐ 'Check list' review for completeness of data<br>☐ Selective review in detail (e.g. stability, specification)<br>☐ Detailed assessment and evaluation report
	Comments:	Click or tap here to enter text.	Click or tap here to enter text.	Click or tap here to enter text.	Click or tap here to enter text.
**Clinical evaluation: factors included in the risk-benefit assessment**	*The clinical opinion takes account of:*	**Type 1**	**Type 2**	**Type 3**	**Priority/fast track products**
	Differences in medical culture/practice	☐ Never<br>☐ Sometimes<br>☐ Always	☐ Never<br>☐ Sometimes<br>☐ Always	☐ Never<br>☐ Sometimes<br>☐ Always	☐ Never<br>☐ Sometimes<br>☐ Always
	Ethnic factors	☐ Never<br>☐ Sometimes<br>☐ Always	☐ Never<br>☐ Sometimes<br>☐ Always	☐ Never<br>☐ Sometimes<br>☐ Always	☐ Never<br>☐ Sometimes<br>☐ Always
	National disease patterns	☐ Never<br>☐ Sometimes<br>☐ Always	☐ Never<br>☐ Sometimes<br>☐ Always	☐ Never<br>☐ Sometimes<br>☐ Always	☐ Never<br>☐ Sometimes<br>☐ Always
	Unmet medical need	☐ Never<br>☐ Sometimes<br>☐ Always	☐ Never<br>☐ Sometimes<br>☐ Always	☐ Never<br>☐ Sometimes<br>☐ Always	☐ Never<br>☐ Sometimes<br>☐ Always

**Additional information, not in the application**	*The agency tries to obtain:*	**Type 1**	**Type 2**	**Type 3**	**Priority/fast track products**
	Other agencies' internal assessment reports	☐ Never<br>☐ Sometimes<br>☐ Always	☐ Never<br>☐ Sometimes<br>☐ Always	☐ Never<br>☐ Sometimes<br>☐ Always	☐ Never<br>☐ Sometimes<br>☐ Always
	Reports available on the Internet (e.g., EPARS)	☐ Never<br>☐ Sometimes<br>☐ Always	☐ Never<br>☐ Sometimes<br>☐ Always	☐ Never<br>☐ Sometimes<br>☐ Always	☐ Never<br>☐ Sometimes<br>☐ Always
	General Internet search	☐ Never<br>☐ Sometimes<br>☐ Always	☐ Never<br>☐ Sometimes<br>☐ Always	☐ Never<br>☐ Sometimes<br>☐ Always	☐ Never<br>☐ Sometimes<br>☐ Always
	Other data (please specify): Click or tap here to enter text.	☐ Never<br>☐ Sometimes<br>☐ Always	☐ Never<br>☐ Sometimes<br>☐ Always	☐ Never<br>☐ Sometimes<br>☐ Always	☐ Never<br>☐ Sometimes<br>☐ Always

PART 3. KEY MILESTONES IN THE REVIEW PROCESS

Review Process Map and Milestones

This part of the questionnaire is based on the **General Model** below, giving a process map and milestones that have been developed from studying procedures followed in 'established' and 'emerging' regulatory agencies. It captures the main steps in the review and approval process and identifies key 'milestone' dates in the process for monitoring and analysing timelines.

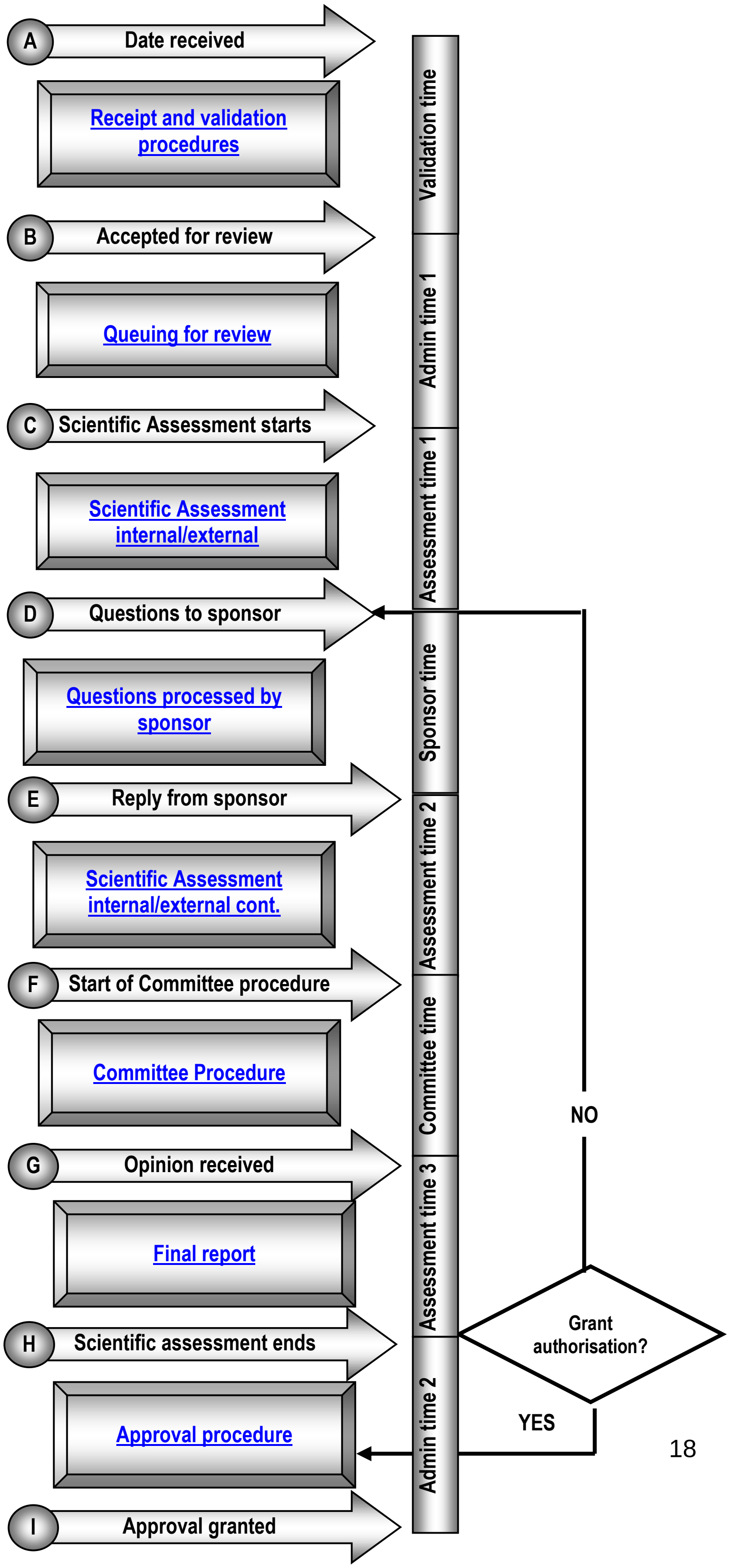

Notes

Receipt and validation may include administrative registration (reference number) and checks on legal requirements, status of company, local agent, manufacturer etc. as well as a 'checklist' validation of the application content (e.g., technical sections, CPP status).

Queuing for review: *Administrative time 1* is a measure of the 'backlog' time (if any) while valid applications wait for action to begin.

Scientific Assessment extends from milestone C to milestone H and is a measure of 'review time.' In some systems, the 'clock' stops when questions are asked and Sponsor ***time*** (milestone D to milestone E) can be measured and deducted from the agency review time.

Questions to sponsor may be batched and sent at one time or asked throughout the review process, in which case the *Sponsor time* is not easily measured.

In some systems, questions may only be sent to the sponsor after the end of the 'first cycle' scientific assessment (at milestone H).

Committee Procedure: Most review procedures for major applications include a step where the opinion of an expert advisory committee is sought. In this scheme, the Committee procedure is 'nested' within the Scientific Assessment but it may take place after the Agency's scientific assessment is complete.

Second cycle: If the application cannot be granted immediately, on technical grounds, it enters a second review cycle (new data point D: questions to sponsor) and a further scientific assessment is made of the additional data. The Committee Procedure may or may not need to be included in the second and subsequent review cycles.

Approval procedure: The time interval after scientific review (*Admin time 2*) while the formal authorisation is issued may be extended by pricing negotiations and finalisation of analytical and GMP checks.

Approval time is measured from milestone A to milestone I.

Review stages and milestones

This section of the questionnaire is based on the ***General Model***.

We recognise that not all systems conform to the ***General Model*** and it would be very helpful if you could provide an outline of the model used by your agency. If this differs according to the **Type of data assessment** (see Part 2. Types of Review Models) please provide information on the different models.

3.1 When information is given on target or actual times please indicate here whether these are counted in:

☐ Calendar days
☐ Working days

3.2 When 'milestone' dates are recorded during the review process is the information entered into an electronic tracking/recording system?

☐ YES, a system is in current use
☐ NO, a system is in development (please specify target date): Click or tap here to enter text.
☐ NO**,** a manual system will be used for the foreseeable future

3.3 Receipt and Validation

Pre-submission requirements

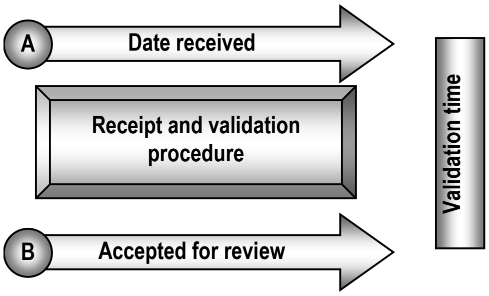

3.3.1 Are there any formal requirements before an application is submitted, for example, notification of intent to submit, assignment of registration code etc.?

☐ NO

☐ YES (please specify): Click or tap here to enter text.

Validation

3.3.2 Is the date of receipt (milestone A) formally recorded?

☐ YES ☐ NO

3.3.3 Are the following administrative items checked in the pre-review validation process?

- Legal status of applicant/local agent: ☐ YES ☐ NO
- GMP status of manufacturer: ☐ YES ☐ NO
- Patent/IP status of active ingredient: ☐ YES ☐ NO

- ○ Whether company has paid the correct fee: ☐ YES ☐ NO
- ○ Other: Click or tap here to enter text.

For those applications where prior authorisation elsewhere is essential (see Part 2 – Types of Review Models) please answer the following questions about the Certificate of a Pharmaceutical Product (CPP):

3.3.4 Is the inclusion of a CPP an absolute requirement before accepting the application as valid?

☐ YES

☐ NO

☐ For some applications (please specify): Click or tap here to enter text.

*3.3.5 **If YES**, must the CPP be legalised by an Embassy or Consulate?*

☐ YES

☐ NO

*3.3.6 **If NO**, please indicate which of the following apply:*

- ○ A CPP must be provided before the authorisation is issued: ☐ YES ☐ NO
- ○ Other evidence of authorisation by other countries is accepted in place of the CPP (e.g., copy of authorisation, Internet reference): ☐ YES ☐ NO

Comments: Click or tap here to enter text.

3.3.7 Is the application also checked for the following items?

- ○ Acceptable format (e.g. ICH CTD or local requirements): ☐ YES ☐ NO
- ○ Correct sections of scientific data (quality, safety, efficacy): ☐ YES ☐ NO
- ○ Other technical items: Click or tap here to enter text.

Acceptance for review/refusal to file

3.3.8 Is the date of acceptance (milestone B) formally recorded?

☐ YES ☐ NO

3.3.9 What happens if the application is incomplete?

☐ Refusal to file: New application must be made

☐ File pending: A request for the missing data is sent to the applicant

*3.3.10 In case of **file pending**, what is the time limit for the applicant to reply?*

Click or tap here to enter text.

Comments: Click or tap here to enter text.

Target time for validation

3.3.11 Is there a target validation time?

☐ YES ☐ NO

*3.3.12 If **YES**, please specify:*

Click or tap here to enter text.

3.4 Queuing/backlog

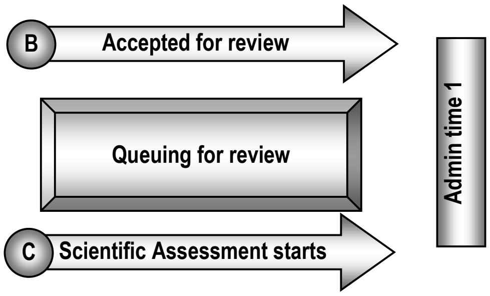

3.4.1 Which of the following applies to the queuing system for new applications?

☐ Held in queue after validation (as in the General Model) after phase 1 validation
☐ Held in queue before validation starts (milestone A)

3.4.2 What is the current queue time (approximately)?

☐ Less than 2 weeks
☐ 2-8 weeks
☐ 2-6 months
☐ 6 months-1 year
☐ More than 1 year

3.4.3 Are priority products taken out of turn in the queuing system?

☐ YES, always
☐ YES, sometimes
☐ NO, all applications await their turn

Comments: Click or tap here to enter text.

3.4.4 Does the agency regard the backlog of applications as a problem?

☐ YES ☐ NO

*3.4.5 If **YES**, how is this being addressed:*

Click or tap here to enter text.

3.5 Scientific Assessment

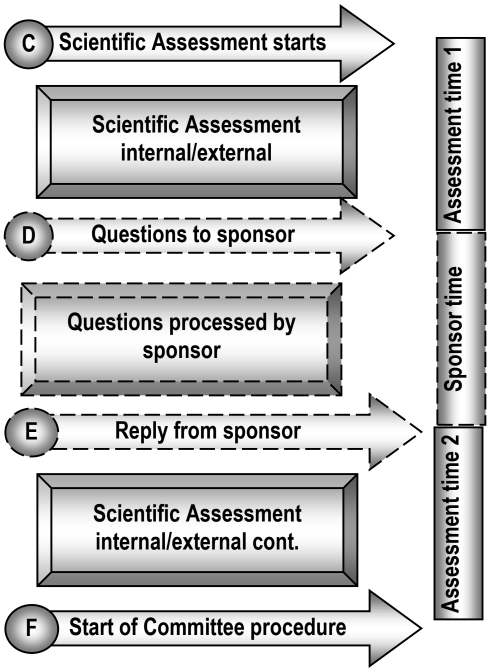

Initiation of scientific review

3.5.1 Is the start of the Scientific Assessment formally recorded (milestone C)?

☐ YES ☐ NO

3.5.2 Is the scientific data separated into three sections (quality, safety, and efficacy) for review?

☐ YES ☐ NO

3.5.3 In what order are the different sections assessed?

☐ In parallel ☐ In sequence

3.5.4 If ***in sequence****, please give order:*

Click or tap here to enter text.

3.5.5 Who carries out the primary scientific assessment?

☐ Agency technical staff
☐ Sent to outside experts
☐ Different procedure for different sections

Please describe the process: Click or tap here to enter text.

Use of outside experts

If outside experts are used for the assessment of scientific data (Milestone C above) please complete the following:

3.5.6 Number of experts on the agency's list or panel:

Click or tap here to enter text.

3.5.7 Main responsibility:

☐ To provide a detailed assessment report and recommendation
☐ To provide a clinical opinion on the product
☐ To provide advice to the agency staff on specific technical issues
☐ Other (Please specify): Click or tap here to enter text.

3.5.8 Is there a contractual agreement on working within deadlines set by the agency?

☐ YES ☐ NO

3.6 Interactions with the Sponsor

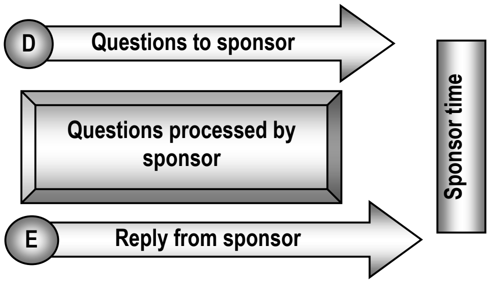

3.6.1 How are questions sent to the Sponsor?

☐ As they arise during the assessment
☐ Collected into a single batch

3.6.2 When are batched questions sent to the Sponsor?

☐ After the initial assessment but before reporting to the Scientific Committee (as in the General model)
☐ Not until the Scientific Committee has given its advice
☐ Before and after reference to the Scientific Committee

3.6.3 Does the scientific review cease while questions are being processed by the Sponsor ('clock stop')?

☐ YES ☐ NO

3.6.4 Can the sponsor time be calculated, i.e., are milestones D and E recorded?

☐ YES ☐ NO

3.6.5 Is the sponsor given a time limit to reply?

☐ YES ☐ NO

3.6.6 If Yes, what time is allowed?

Click or tap here to enter text.

Meetings

3.6.7 Can the Sponsor hold meetings with the agency staff to discuss questions and queries that arise during the assessment?

☐ YES ☐ NO

*3.6.8 If **Yes**, what conditions and restrictions (if any) are applied:*

Click or tap here to enter text.

3.6.9 Can the Sponsor hold meetings with the agency staff to discuss questions and queries that arise during the assessment?

☐ YES ☐ NO

*3.6.10 **If Yes**, what conditions and restrictions (if any) are applied:*

Click or tap here to enter text.

3.7 Review by Scientific Committee(s)

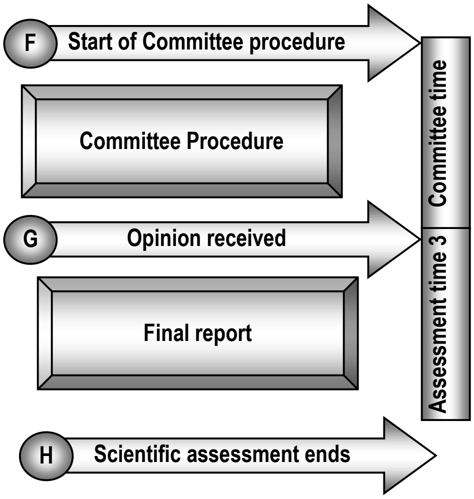

3.7.1 Is a Committee of Experts (internal and/or external) used in the review process?

☐ YES ☐ NO

3.7.2 If YES, at which stage in the review?

☐ Responsible for the whole assessment of the dossier from the start of the review
☐ Integrated into the agency's own internal/external scientific review procedure
☐ Consulted after the agency has reviewed and reported on the scientific data
☐ Other (Please specify): Click or tap here to enter text.

3.7.3 Are the dates at the start and end of the Committee Review recorded (milestones F and G)?

☐ YES ☐ NO

3.7.4 Is the agency mandated to follow the Committee recommendation?

☐ YES ☐ NO

3.7.5 Is there a time limit for the Committee Procedure?

☐ YES ☐ NO

*3.7.6 **If YES**, please give the target:*

Click or tap here to enter text.

*3.7.7 **If NO**, what is the time range?*

Click or tap here to enter text.

3.7.8 Is there an additional step in the scientific review process, after the Committee has given its opinion?

☐ YES ☐ NO

3.7.9 If YES, please describe briefly the work carried out at this stage (e.g., final report and agency opinion):

Click or tap here to enter text.

3.7.10 If NO, the milestone G will mark the end of the scientific review for the purpose of calculating the review time:

Click or tap here to enter text.

Target timelines for the review process

3.7.11 Is a target time set for the scientific review (milestones C to H)?

☐ YES ☐ NO

*3.7.12 If **YES** please give target*

Click or tap here to enter text.

3.8 Recommendation on the Application

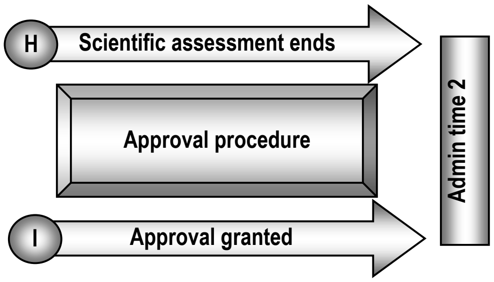

At the end of the Scientific Review (see General Model) there is normally recommendation that either:

- The product meets the scientific criteria for authorisation (proceed to approval procedure) *or*
- Further data is required before the scientific criteria are met (application enters a **second cycle** at milestone D (questions to Sponsor) *or*
- The application should be refused (not shown in the General Model)

Responsibility for the authorisation decision

3.8.1 Who makes the decision that a marketing authorisation can be granted?

☐ The Scientific Advice Committee
☐ The Head of the Agency
☐ The Minister of Health
☐ Other (please specify): Click or tap here to enter text.

3.8.2 If Scientific Advice Committee is used as per 3.8.1, what kind of decision-making process is used?

☐ Consensus process by the Committee
☐ Majority vote by the Committee
☐ One individual makes the final decision based on the Committee recommendations
☐ Other (please specify): Click or tap here to enter text.

Other criteria to be met

*3.8.3 Is the issue of the authorisation dependent on a **pricing agreement**?*

☐ YES ☐ NO

*3.8.4 **If YES**, when are the pricing negotiations started?*

☐ At the start of the scientific review
☐ After the end of the scientific review
☐ After the start but before the end of the scientific review

*3.8.5 Is the issue of the authorisation dependent on **sample analysis**?*

☐ YES ☐ NO

*3.8.6 **If YES**, when is the analytical work started?*

☐ In parallel with the scientific review
☐ At the end of the scientific review
☐ After the start, but before the end of the scientific review

*3.8.7 Is there a separate **negotiation of the product labelling/product information** after the scientific opinion is given but before the approval is issued?*

☐ YES ☐ NO

Comments: Click or tap here to enter text.

3.8.8 Please specify any other legal/administrative matters that must be finalised before the approval can be issued:

Click or tap here to enter text.

3.8.9 Is the sponsor informed of a positive scientific opinion at milestone G, i.e., before the authorisation is issued?

☐ YES ☐ NO

3.8.10 Approximately how long does it take from receiving a positive scientific opinion (at milestone H) to issuing an approval (milestone I)?

☐ Less than a month
☐ 1-3 months
☐ 3-6 months
☐ Over 6 months
Comments: Click or tap here to enter text.

3.9 Metrics on the Approval Process

It would be very helpful to have the following information on processing times for marketing authorisations that have been received and/or determined in the three years:

*3.9.1 **Actual approval times (average)***

Type	Time from receipt of application to issue of approval		
	**2018**	**2019**	**2020**
New Active Substances approved	Click or tap here to enter text.	Click or tap here to enter text.	Click or tap here to enter text.
Major Line Extensions approved	Click or tap here to enter text.	Click or tap here to enter text.	Click or tap here to enter text.
Generics approved	Click or tap here to enter text.	Click or tap here to enter text.	Click or tap here to enter text.
WHO Pre-qualified generics approved	Click or tap here to enter text.	Click or tap here to enter text.	Click or tap here to enter text.

PART 4. GOOD REVIEW PRACTICES (GRevP): BUILDING QUALITY INTO THE REVIEW PROCESS

Quality in the assessment and registration process is important to regulatory authorities as it ensures consistency, transparency, timeliness and competency in the review processes. Regulatory authorities are continuously developing and implementing a variety of measures to improve and achieve higher quality standards and to meet the expectations of industry and the general public. The purpose of this section of the questionnaire is to obtain an insight into the strategies, measures and resources that agencies have in place to develop and maintain quality in their review processes.

4.1 *General measures used to achieve quality*

Please indicate the quality measures currently in place and, where there are none, what, if any, plans there are to introduce such measures in the foreseeable future.

Good Review Practices (GRevP)

"*A code about the process and the documentation of review procedures that aims to standardise and improve the overall documentation and ensure timeliness, predictability, consistency and high quality of reviews and review reports"* (see Glossary).

4.1.1 How does your agency define GRevP: Is it different from the Glossary?

☐ YES ☐ NO

*4.1.2. **If different**, please define here:*

Click or tap here to enter text.

4.1.3 Please outline the key elements that make up GRevP in your agency:

Click or tap here to enter text.

4.1.4 Has the agency formally or informally implemented GRevP?

☐ YES (Formally)
☐ YES (Informally)
☐ NO

*4.1.5 **If YES**, please give the title and date of formal implementation:*

Click or tap here to enter text.

4.1.6 How has this been implemented? (Please select the appropriate box(s)):

☐ Guidelines
☐ Standard Operating Procedure (SOP)
☐ GRevP Training Program
☐ Other (Please specify): Click or tap here to enter text.

4.1.7 Are these documents open and available to the public?

☐ YES ☐ NO

*4.1.8 **If YES**, please describe how:*

Click or tap here to enter text.

4.1.9 Are these documents open and available to the public?

☐ YES ☐ NO

*4.1.10 **If YES**, please describe how:*

Click or tap here to enter text.

4.1.11 Was the establishment of your GRevP based on other agencies or International standards?

☐ YES ☐ NO

*4.1.12 **If YES,** please state the name of the agency(ies)/ or internationals standards on which your GRevP has been based:*

Click or tap here to enter text.

4.1.13 Are you satisfied with your existing GRevP framework?

☐ Satisfied
☐ Could be improved
☐ Unsatisfied

*4.1.14 **If could be improved or unsatisfied**, please select the reason(s) that best describes your situation:*

☐ System still evolving
☐ Requires additional training to understand and learn about Good Review Practice
☐ Poor acceptance/utilization by staff
☐ Benefits of implementing GRevP are not apparent so far
☐ Other (please provide details): Click or tap here to enter text.

4.1.15 If you do not have a formal GRevP system in place are there plans to establish this within the next two years?

☐ YES ☐ NO

Internal Quality Policy

"*Overall intentions and direction of an organisation related to quality as formally expressed by top management*" (see Glossary).

4.1.16 Does the agency have an Internal Quality Policy?

☒ YES ☐ NO

*4.1.17 **If NO**, are there plans to establish this within the next two years?*

☐ YES ☐ NO

SOPs

"*SOPs (Standard Operating Procedures) are written documents that describe in detail the routine procedures to be followed for a specific operation*" (see Glossary).

4.1.18 Are there SOPs for the guidance of scientific assessors?

☒ YES ☐ NO

*4.1.19 **If NO**, are there plans to establish SOPs within the next two years?*

☐ YES ☐ NO

4.1.20 Are there SOPs for the advisory committee consulted during the review process?

☐ YES
☐ NO
☐ No committee

*4.1.21 **If NO**, are there plans to establish SOPs within the next two years?*

☐ YES ☐ NO

4.1.22 Are SOPs used for any other procedures in the regulatory review process (e.g., validation)?

☐ YES, please specify: Click or tap here to enter text.
☐ NO

Assessment Templates

"set out the content and format of written reports on scientific reviews" (see Glossary).

4.1.23 Are there Assessment Templates for reports on the scientific review of an NAS?

☐ YES ☐ NO

*4.1.24 **If NO**, are there plans to establish this within the next two years?*

☐ YES ☐ NO

*4.1.25 **If YES**, are these based on another agency's assessment template?*

☐ YES, please specify which agency(ies): Click or tap here to enter text.

☐ NO

4.1.26 Is there an SOP for completing an assessment template?

☐ YES ☐ NO

4.1.27 Select which elements from the list below are included in your agency assessment template:

☐ Drug Substance
☐ Drug Product
☐ Comments on label
☐ Non-clinical GLP Aspects
☐ Non-clinical Pharmacokinetic
☐ Toxicology
☐ Regulatory background (worldwide status on regulatory agencies)
☐ GCP aspects
☐ Clinical Pharmacology (PK & PD)
☐ Clinical Efficacy
☐ Clinical Safety
☐ List of questions for sponsors
☐ Benefit Risk Reduction
☐ Ethnic factors (e.g., consideration of bridging studies)
☐ Other (please specify): Click or tap here to enter text.

4.1.28 Would the agency be open to sharing their assessment template or points to consider with CIRS?

☐ YES ☐ NO

Assessment report

4.1.29 Do you produce an assessment report (AR) following the review?

☐ YES ☐ NO

*4.1.30 **If YES**, is there an SOP for completing the AR?*

☐ YES ☐ NO

4.1.31 What language is the AR prepared in?

☐ Local language
☐ English

4.1.32 Do you share your AR with other regulatory authorities?

☐ YES
☐ NO
☐ Sometimes

*4.1.33 Do you put your **full** AR on the website?*

☐ YES
☐ NO
☐ Sometimes

*4.1.34 Do you put your **abridged** AR on the website?*

☐ YES
☐ NO
☐ Sometimes

*4.1.35 Do sponsors get a copy of the **full** assessment report?*

☐ YES ☐ NO

4.1.36 Do sponsors have any involvement in the following in relation to AR:

☐ Preparation of assessment reports
☐ Comments on the assessment reports
☐ Translation of assessment reports
☐ Distribution of assessment reports

Peer Review

"*is an additional evaluation of an original assessment that is carried out by an independent person or committee. Peer review can occur either during assessment of a dossier or at the time of sign-off*" (see Glossary).

*4.1.37 Are **external** peer reviews carried out when a NAS is assessed?*

☐ YES ☐ NO

*4.1.38 **If NO**, are there plans to introduce these within the next two years?*

☐ YES ☐ NO

*4.1.39 Are **internal** peer reviews carried out when a NAS is assessed?*

☒ YES ☐ NO

*4.1.40 **If NO**, are there plans to introduce these within the next two years?*

☐ YES ☐ NO

4.1.41 Are there other general procedures in place to monitor the quality of the review process?

☐ YES ☐ NO

4.1.42 What other tools does your agency use to build quality into the assessment process? (e.g., Internal procedure could include: quality assurance and quality control meeting; stakeholder meeting; channel for grievance; survey of performance from sponsors)

Click or tap here to enter text.

4.2 Quality Management

Reasons for introducing quality measures in the agency

4.2.1 From the following list, please select the three most important reasons for the introduction of quality measures:

☐ To be more efficient
☐ To ensure consistency
☐ To achieve stakeholder satisfaction

☐ To improve predictability
☐ To minimise errors
☐ To increase transparency
☐ To improve communications in the agency
☐ To allocate the regulatory resources
☐ Other (please specify): Click or tap here to enter text.

Monitoring to improve quality

4.2.2 Which of the following activities are undertaken by the agency to bring about continuous improvement in the assessment and registration process?

☐ Reviewing assessors' feedback and taking necessary action
☐ Reviewing stakeholders' feedback (e.g. through complaints, meetings or workshops) and taking necessary action
☐ Using an internal tracking system to monitor (e.g. consistency, timeliness, efficiency and accuracy)
☐ Carrying out internal quality audits (e.g. self-assessments) and using findings to improve the system
☐ Having external quality audits by an accredited certification body to improve the system
☐ Having a 'post approval' discussion with the sponsor to provide feedback on the quality of the dossier and obtain the company's comments

Management responsibility for quality

4.2.3 Does the agency have a dedicated department for assessing and/or ensuring quality in the assessment and registration process?

☐ YES ☐ NO

*4.2.4. **If YES**, how many staff are involved?*

Click or tap here to enter text.

4.2.5 How often do you assess and/or ensure quality in the assessment and registration process?

☐ Annually
☐ Semi-annually
☐ Ad hoc
☐ Other, please specify: Click or tap here to enter text.

4.2.6 To whom does this section report (e.g., the Chief Executive Officer of the agency)?

Click or tap here to enter text.

*4.2.7 **If NO** to 4.2.3, is the agency thinking of setting up such a department?*

☐ YES ☐ NO

4.3 Quality in the Review and Assessment Process

Improving the quality of applications

4.3.1 Does the agency have official guidelines to assist industry in the registration of medicinal products?
☐ YES ☐ NO

4.3.2 ***If YES,*** *how are these guidelines made available? (Please indicate all that apply)*

☐ Through the agency's website
☐ Through official publications
☐ On request
☐ Through Industry associations
☐ Other, please specify: Click or tap here to enter text.

4.3.3 What language/s are the guidelines available in?

☐ Local language only
☐ English
☐ Other, please specify: Click or tap here to enter text.

Improving quality through interactions with applicants

4.3.4 Does the agency provide pre-submission scientific advice to applicants?

☐ YES ☐ NO

4.3.5 ***If YES,*** *how is the quality of that advice monitored?*

Click or tap here to enter text.

4.3.6 Is the applicant given details of technical staff that can be contacted to discuss an application during review?

☐ YES ☐ NO

4.3.4 Please indicate which of the following best describes the level of contact that companies have with agency staff or outside experts during development and during the agency's assessment:

	**Development**	**Assessment**
• Extensive formal contact (including scheduled meetings)	☐	☐
• Extensive informal contact (frequent telephone or email contact)	☐	☐
• Some formal contact (possibility of meetings)	☐	☐
• Some informal contact (possibility of telephone or email contact)	☐	☐
• None, or minimal formal contact (rare occurrences of contact, via letter or fax)	☐	☐
• None, or minimal informal contact (rare telephone or email contact)	☐	☐

4.3.5 Please comment on general policy for contact with applicants:

Click or tap here to enter text.

Scientific Committee Procedures

If your review procedure includes obtaining the advice of a scientific committee of internal and/or external experts (as in Section Review by Scientific Committee) please complete the following:

4.3.6 Name of the Committee :

Click or tap here to enter text.

4.3.7 Number of Committee members :

Click or tap here to enter text.

4.3.8 How frequently does the Committee meet?

☐ Once a week
☐ Once a month
☐ Other, please specify: Click or tap here to enter text.

4.3.9 For NAS applications and major line extensions does the Committee review:

☐ All applications
☐ Selected dossiers, please specify: Click or tap here to enter text.

4.3.10 Does the Committee review:

☐ The complete dossier
☐ Assessment reports from the reviewers

Shared and Joint reviews with other Regulatory Agencies outside of your country

A ***shared review*** is "*one where each participating agency takes responsibility for reviewing a separate part of the dossier*". A ***joint review*** is "*one where the whole dossier is reviewed by each agency and the outcome is discussed before a decision is taken*" (see Glossary).

4.3.11 Is your agency part of any regional alignment initiatives?

☐ YES ☐ NO

*4.3.12 **If YES**, please specify and complete Appendix II:*

Click or tap here to enter text.

4.3.13 Are bilateral/multilateral information sharing agreements in place with other jurisdictions?

☐ YES ☐ NO

*4.3.14 **If YES**, what is the general nature of those agreements?*

Click or tap here to enter text.

4.3.14 Does your agency conduct shared or joint reviews with other regulatory authorities?

☐ YES, regularly. Please state which authorities: Click or tap here to enter text.

☐ YES, occasionally. Please state which authorities: Click or tap here to enter text.
☐ NO, this has never been undertaken

4.3.15 If YES, do you have formal measures in place to ensure consistent quality during the review?

☐ YES ☐ NO

*4.3.16 If **YES**, please specify:*

Click or tap here to enter text.

*4.3.17 If **NO**, do you anticipate undertaking such reviews within the next two years?*

☐ YES ☐ NO

4.3.18 Have these joint reviews influenced the way in which your agency conducts reviews in general?

☐ YES, please specify: Click or tap here to enter text.
☐ NO

4.4 Training and continuing education as an element of quality

The following questions relate to training and continuing education of assessors working within the agency, including those employed on a full-time basis and those contracted for specific assessments were necessary.

4.4.1 Do you have a formal training programme for assessors?

☐ YES ☐ NO

4.4.2 Which of the following methods are used for training assessors?

☐ Induction training
☐ On job training
☐ External courses
☐ Post-graduate degrees
☐ Placements and secondments in other regulatory authorities
☐ External speakers invited to the agency
☐ Participation in international workshops/ conferences
☐ In-house courses
☐ Other, please specify: Click or tap here to enter text.

4.4.3 Do you have a formal training programme for assessors?

☐ YES ☐ NO

Collaboration with other agencies

4.4.4 Does your agency seek direct assistance of more experienced agencies for development of SOPs and Guidelines?

☐ YES ☐ NO

*4.4.5 If **YES,** please give details:*

Click or tap here to enter text.

4.4.6 Does your agency mainly develop SOP, Guidelines etc., based on information published by more experienced agencies:

☐ YES ☐ NO

4.4.7 Does your agency collaborate with other agencies in the training of assessors?

☐ YES, please specify: Click or tap here to enter text.
☐ NO

Completion of training

4.4.8 Is training tested in examination situations once completed?

☐ YES
☐ NO
☐ Partly

4.4.9 Is completion of training courses required for professional advancement?

☐ YES
☐ NO
☐ Partly

4.5 Transparency of the review process

This section examines 'transparency' in terms of the ability and willingness of the agency to assign time and resources to providing information on its activities to both the informed public (which includes health professionals) and industry.

4.5.1 What priority does your agency assign to being open and transparent in relationships with the public, professions and industry?

☐ High priority
☐ Medium priority
☐ Low priority
Please comment: Click or tap here to enter text.

4.5.2 What are the main drivers for establishing transparency? Please indicate the top three incentives for assigning resources to activities that enhance the openness of the regulatory system:

☐ Political will
☐ Public pressure
☐ Press and media attention
☐ Need to increase confidence in the system
☐ Need to provide assurances on safety safeguards
☐ Better staff morale and performance
☐ Other, please specify: Click or tap here to enter text.

Transparency to the public

The following questions explore the availability of information to the general public on the performance of regulatory authorities.

4.5.3 Please indicate which of the following information items about the assessment and registration of marketing applications is available to the public:

☐ Approval of products
☐ Approval times
☐ Summary of the grounds on which the approval was granted
☐ Advisory Committee meeting dates
☐ Other, please specify: Click or tap here to enter text.

4.5.4 How is this information made available?

☐ Official journal/periodical publication
☐ From an official Internet website
☐ On request
☐ Other, please specify: Click or tap here to enter text.

Transparency to companies on the application progress

4.5.5 Are companies able to follow the progress of their own applications?

☐ YES ☐ NO

4.5.6 If YES, please indicate the mechanisms available to industry:

☐ Telephone contact
☐ Electronic access to the status of applications
☐ E-mail contact
☐ Other, please specify Click or tap here to enter text.

4.5.7 Are companies given detailed reasons for rejection of an application for registration?

☐ YES ☐ NO

Facilities for providing information

4.5.8 Is there an electronic system for registering and tracking applications?

☐ YES ☐ NO

*4.5.9 **If YES**, please indicate whether it has the following capabilities:*

☐ Tracking applications that are under review and identifying the stage in the process
☐ Signalling that target review dates have been exceeded
☐ Recording the terms of the authorisation once granted
☐ Archiving information on applications in a way that can be searched

*4.5.10 **If NO,** are there plans to introduce such a system?*

☐ YES ☐ NO

*4.5.11 **If so**, please give target date for implementation:*

Click or tap here to enter text.

PART 5. QUALITY DECISION-MAKING PROCESSES

Regulatory agencies consider various types of information needed to carry out their assessment of new medicines, but it is not always clear how the decisions, which require human judgment and interpretation, are made around the data. According to the well-established principles of the science of decision making, any organisation that seeks to improve its productivity and consistency should also routinely measure the quality of its decision-making process. These questions aim to uncover the decision-making practices of your agency, focusing on the process to approve or reject a New Drug Application.

5.1 Decision-making frameworks

A Framework is "*a set of principles, guidelines and tools which provide a structured systematic approach to guide decision-makers in selecting, organising, understanding and summarising subjective values and judgments that form the basis of a decision, as well as communicating the evidence relevant to the decision*" (see Glossary).

5.1.1 Does your agency have a framework in place that forms the basis of the decision to approve or reject a New Drug Application (NDA)?

☐ YES ☐ NO

If "No", please answer 5.1.2-5.1.3, and then go to 5.2, if "Yes", please go to section 5.1.4 and continue

5.1.2 Why a framework is not used? (mark all that apply)

☐ Lack of a validated framework
☐ Lack of knowledge/training on decision making in general
☐ Benefits of a framework not apparent
☐ Resource/administrative limitation
☐ Others, please specify: Click or tap here to enter text.

5.1.3 Are there plans to adopt a framework in the next two years?

☐ YES
☐ NO
☐ Not sure

5.1.4 Which statement best describes the nature of your framework?

☐ The framework has been formally defined and codified
☐ The framework is informal, by custom and practice (i.e. it has never been clearly agreed but over time has become the process)

5.1.5 In your view, which Quality Decision-Making Practices have been implemented into your agency's framework (to approve/reject an NDA) and are they adhered to in practice?

See the Appendix I for explanation on the Practices.

Practice	Implemented into framework (select one)			Adhered to in practice (select one)		
	Fully	Partially	Not	Fully	Partially	Not
1. Have a systematic, structured approach (consistent predictable and timely)	☐	☐	☐	☐	☐	☐
2. Assign clear roles and responsibilities (decision makers, advisors, information providers)	☐	☐	☐	☐	☐	☐
3. Assign values and relative importance to decision criteria	☐	☐	☐	☐	☐	☐
4. Evaluate both internal and external influences/biases	☐	☐	☐	☐	☐	☐
5. Examine alternative solutions	☐	☐	☐	☐	☐	☐
6. Consider uncertainty	☐	☐	☐	☐	☐	☐
7. Re-evaluate as new information becomes available	☐	☐	☐	☐	☐	☐
8. Perform impact analysis of the decision	☐	☐	☐	☐	☐	☐
9. Ensure transparency and provide a record trail	☐	☐	☐	☐	☐	☐
10. Effectively communicate the basis of the decision	☐	☐	☐	☐	☐	☐

5.1.6. Please comment and provide examples

Click or tap here to enter text.

5.2 Decision-making challenges

5.2.1 In your opinion, does your agency have measures in place to minimise impact of subjective influences / biases on your agency's decision making for the process to approve/reject an NDA.

Please see the Glossary for more explanation on biases.

☐ YES ☐ NO

Comment: Click or tap here to enter text.

5.2.2 Are there formal assessments in place to periodically measure the quality of decision-making within your agency for the process to approve/reject an NDA?

☐ Yes, and this is to measure the quality of the process of decision making
☐ Yes, and this is to measure the quality of the outcome
☐ No

Comment: Click or tap here to enter text.

5.2.3 Does your agency provide training in the area of quality decision making?

☐ YES ☐ NO

Comment: Click or tap here to enter text.

5.2.4 Do you think that your agency's decision-making process for approving/rejecting an NDA could be improved?

☐ YES ☐ NO

Comment: **Click or tap here to enter text.**

PART 6. CONCLUDING OBSERVATIONS

The purpose of the following two questions is to try to identify the Agency's own perception of its unique positive qualities and the major impediments it faces in carrying out the review of new medicines and making them available to meet patients' needs.

6.1 List three factors that make a major contribution to the effectiveness and efficiency of your agency's review procedures and decision-making processes for NAS applications:

1. Click or tap here to enter text.
2. Click or tap here to enter text.
3. Click or tap here to enter text.

6.2 List three factors that act as barriers to making new medicines available in a timely manner through the regulatory process:

1. Click or tap here to enter text.
2. Click or tap here to enter text.
3. Click or tap here to enter text.

6.3 Are there any important documents related to GRevP that you would like to share with CIRS?

☐ YES ☐ NO

6.4 If yes please list and provide directly to CIRS:

Click or tap here to enter text.

ACKNOWLEDGEMENT

Name of person completing questionnaire:
Title:
Date:

<u>Thank you for completing this questionnaire</u>

GLOSSARY AND ABBREVIATIONS

**Additional information**	Additional data or additional analyses of existing data requested from the sponsor by the regulatory agency during the review process.
**Advisory Committee**	An expert committee that advises the regulatory agency of the safety, quality and efficacy of new medicines for human use.
**Approval**	The approval of a drug product by a regulatory agency, signified by the granting of a marketing authorisation, or the issue of a technical approval letter. However, the product may still not be marketable until negotiations for pricing and reimbursement are concluded.
**Assessment template**	Set out the content and format of written reports on scientific reviews
**Bias**	A subjective influence. Different types have been identified for example:<br>· Action-oriented influences drive us to take action less thoughtfully than we should e.g. Excessive optimism, overconfidence, gut-feeling<br>· Interest influences arise in the presence of conflicting incentives and even purely emotional ones. E.g. misaligned individual incentives and attachments<br>· Pattern-recognition influences lead us to recognize patterns even where there are none e.g. confirmation bias to seek out information that supports a favoured decision<br>· Stability influences create a tendency toward inertia in the presence of uncertainty e.g. preference for the status quo in the absence of pressure to change it<br>*Source: Lovallo and Sibony*
**Certificate of Pharmaceutical Product (CPP)**	Certificate issued in the format recommended by the World Health Organization (WHO), which establishes the status of the pharmaceutical product and of the applicant for this certificate in the exporting country.
**Chemistry, manufacturing and controls (CMC)**	All activities conducted to optimize, scale-up and validate the processes and technologies for transfer to manufacture and all Quality Assurance (QA), Quality Control (QC) and Chemistry, manufacturing and controls support activities (e.g. CMC project management including CMC contribution to project teams). This includes all drug substance R&D i.e. process research and process development, all drug product R&D i.e. formulation development and process development, all analytical work for drug substance R&D and drug product R&D, clinical supplies and CMC's involvement in the compilation of regulatory documentation.
**Clinical summary**	Summary of clinical study data that typically includes biopharmaceutic studies and associated analytical methods, clinical pharmacology studies, clinical efficacy, clinical safety, literature references, and synopses of individual studies. Refers to Module 2.7 in CTD format.

**Common Technical Document (CTD) format**	Common technical document (CTD) as outlined in the ICH guideline M4 (Organisation of the common technical document for the registration of pharmaceuticals for human use; M4).
**Framework**	A set of principles, guidelines and tools which provide a structured systematic approach to guide decision-makers in selecting, organising, understanding and summarising subjective values and judgments that form the basis of a decision, as well as communicating the evidence relevant to the decision
**Good Clinical Practice (GCP)**	An international ethical and scientific quality standard for designing, conducting, recording and reporting trials that involve the participation of human subjects. It aims to provide a unified standard for the ICH regions to facilitate the mutual acceptance of clinical data by the regulatory authorities in these jurisdictions.
**Good Review Practices (GRevP)**	A code about the process and the documentation of review procedures that aims to standardise and improve the overall documentation and ensure timeliness, predictability, consistency and high quality of reviews and review reports.
**Internal reviewers**	Internal reviewers are employees of the agency
**International Conference on Harmonisation (ICH)**	Brings together the regulatory authorities and pharmaceutical industry to discuss scientific and technical aspects of drug registration.
**Joint review**	The whole dossier is reviewed by each agency and the outcome is discussed before a decision is taken.
**Major Line Extension (MLE)**	A major line extension is a modification to an authorised Medicinal Product that is sufficiently great that it cannot be considered to be a simple variation to the original product, but requires a new product authorisation. Such modifications include major new therapeutic indications or new disease states, extension to new patient populations (e.g., paediatrics), a new route of administration or a novel drug delivery system.
**Marketing Authorisation**	Authorisation issued by a regulatory to launch a drug product on the market.
**Marketing Authorisation Application (MAA)**	Authorisation application submitted to a regulatory agency to launch a drug product on the market to which the application has been submitted
**Milestone**	A milestone must involve some form of dated written document to which the regulatory agency can refer. In addition, a milestone must be considered by the regulatory agency to be the point at which one event stops and the next one begins so that the times for events are interdependent.
**New Active Substance (NAS)**	A new chemical, biological or pharmaceutical active substance includes:<br>· a chemical, biological or radiopharmaceutical substance not previously authorised as a medicinal product;<br>· an isomer, mixture of isomers, a complex or derivative or salt of a chemical substance not previously authorised as a medicinal product but differing in properties with regard to safety and efficacy from that chemical substance previously authorised;<br>· a biological substance previously authorised as a medicinal product, but differing in molecular structure, nature of the source material or manufacturing process;

	· a radiopharmaceutical substance which is radionucleotide, or a ligand not previously authorised as a medicinal product, or the coupling mechanism to link the molecule and the radionucleotide has not been previously authorised.
**Non-clinical summary**	Summary of non-clinical data including: pharmacology, pharmacokinetics and toxicology. Refers to Module 2.6 in CTD format.
**Peer review**	Peer review means an additional evaluation of an original assessment carried out by an independent person or committee. Peer review can occur either during assessment of a dossier, or at sign-off.
**Quality control (QC)**	Quality control is operational techniques and activities that are used to fulfil requirements for quality. It involves techniques that monitor a process and eliminate causes of unsatisfactory performance at all stages of the quality cycle.
**Quality policy**	Overall intentions and direction of an organisation related to quality as formally expressed by top management.
**Questions to sponsor**	The process of asking the sponsor for additional data or additional analyses of existing data. The requests are made by the regulatory agency during the review process.
**Scientific assessment**	Review of the dossier in terms of safety, quality and efficacy of data submitted.
**Shared review**	Each agency takes responsibility for assessing a separate part of a dossier.
**Sponsor**	A company, person, organisation or institution that takes responsibility for initiating, managing or financing a clinical study.
**Standard Operating Procedures (SOPs)**	Detailed, written instructions to achieve uniformity of the performance of a specific function
**Validation of a dossier**	The process whereby the agency verifies that all parts of the submitted dossier are present and complete and suitable to be assessed as part of the assessment and registration process.

APPENDIX I – QUALITY DECISION-MAKING PRACTICES

Transparency • Predictability • Consistency

Development of the 10 Quality Decision-Making Practices

As a result of the discussion from CIRS Workshops in June 2015 and February 2016[3], the following Guidance Notes were produced to describe the 10 QDMPs in more detail.

QDMP 1. Have a systematic, structured approach to aid decision making (consistent, predictable and timely)

- *Establish the decision context, objectives and assumptions made.*
- *Employ frameworks, guidelines and tools for structuring the decision-making process.*
- *Such an approach should ensure that the process is systematic, which in turn would enable better consistency compared with similar past decisions, as well as predictability and timeliness.*

QDMP 2. Assign clear roles and responsibilities (decision makers, advisors, information providers)

- *The roles and responsibilities should be clearly defined in terms of individuals who provide information (including external input), compared with those who advise on the decision or make the final decision.*
- *The roles and responsibilities of each stakeholder (regulatory authorities, HTA agencies and companies) should be transparent and well communicated, which should help manage expectations.*

QDMP 3. Assign values and relative importance to decision criteria

- *The relevant criteria for the decision must be determined to ensure that these are in line with the decision context and overall objective. The criteria should be weighted, for example, by ranking or rating their relative importance.*

QDMP 4. Evaluate both internal and external influences/biases

- *Stakeholders need to be aware of personal considerations, subjective influences and biases, acknowledge them and minimise where possible. Potential biases that need to be considered[4]:*
  - *Action-oriented bias: excessive optimism, overconfidence in own judgement and gut-feeling*
  - *Interest-oriented bias: inappropriate attachments and misaligned incentives*
  - *Pattern recognition: generalising based on recent events and seeking out information that supports a favoured decision, which could lead to perpetuating previous mistakes*
  - *Stability bias: preference for status quo and tendency for inertia in the presence of uncertainty*

QDMP 5. Examine alternative solutions

- *Decision makers should actively explore possible options during the decision-making process.*
- *The alternatives need to be assessed, for example using a SWOT analysis, against the relevant decision criteria in order to determine the best outcome.*

QDMP 6. Consider uncertainty

- *The extent and limitations of available information need to be judged for each decision criterion in relation to the alternative options.*
- *Stakeholders must be explicit regarding acceptability of benefits and harms and how this affects their approach.*

QDMP 7. Re-evaluate as new information becomes available

- *This should be actively carried out at all stages during the lifecycle of medicines' development.*
- *This may be a safeguard against plunging in or procrastination and/or perpetuating previous mistakes as well as identifying cultural/organisational/hierarchical influences (e.g. individual vs. organisational, group successes and group failures).*

QDMP 8. Perform impact analysis of the decision

- *The impact of the decision needs to be considered on both internal and external stakeholders.*
- *The analysis must relate to present situation, but also to the future and should take into account elements of quality/validity of data, political/financial/competitor influences and procedures for similar decisions.*

QDMP 9. Ensure transparency and provide a record trail

- *It must be clear how the decision was made and details must be consistently documented in a manner that can be easily followed or audited by appropriate stakeholders.*

QDMP 10. Effectively communicate the basis of the decision

- *The basis of the decision needs to be appropriately communicated to the relevant stakeholders, both internally and externally.*

---

[3] The Centre for Innovation in Regulatory Science. *Publications.* Available at: http://www.cirsci.org/past-workshops-and-publications/

[4] Lovallo D, Sibony O. *The case of behavioral strategy.* McKinsey Quarterly. Available at: http://www.mckinsey.com/insights/ strategy/ the_case_for_behavioral_strategy.
